# Prevalence of Nelson’s syndrome after bilateral adrenalectomy in patients with cushing’s disease: a systematic review and meta-analysis

**DOI:** 10.1007/s11102-021-01158-z

**Published:** 2021-05-25

**Authors:** Eleni Papakokkinou, Marta Piasecka, Hanne Krage Carlsen, Dimitrios Chantzichristos, Daniel S. Olsson, Per Dahlqvist, Maria Petersson, Katarina Berinder, Sophie Bensing, Charlotte Höybye, Britt Edén Engström, Pia Burman, Cecilia Follin, David Petranek, Eva Marie Erfurth, Jeanette Wahlberg, Bertil Ekman, Anna-Karin Åkerman, Erik Schwarcz, Gudmundur Johannsson, Henrik Falhammar, Oskar Ragnarsson

**Affiliations:** 1 Department of Internal Medicine and Clinical Nutrition, Institute of Medicine at Sahlgrenska Academy, University of Gothenburg, 413 45 Gothenburg, Sweden; 2 The Department of Endocrinology, Sahlgrenska University Hospital, Blå stråket 5, 413 45 Gothenburg, Sweden; 3 Department of Environmental and Occupational Health School of Public Health and Community Medicine, University of Gothenburg, 4053 Gothenburg, Sweden; 4 Department of Public Health and Clinical Medicine, Umeå University, 901 87 Umeå, Sweden; 5 Department of Molecular Medicine and Surgery, Karolinska Institutet, 17176 Stockholm, Sweden; 6 Department of Endocrinology, Karolinska University Hospital, 171 76 Stockholm, Sweden; 7 Department of Endocrinology and Diabetes, Uppsala University Hospital, and Department of Medical Sciences, Endocrinology and Mineral Metabolism, Uppsala University, 751 85 Uppsala, Sweden; 8 Department of Endocrinology, Skåne University Hospital, University of Lund, 205 02 Malmö, Sweden; 9 Department of Endocrinology, Skåne University Hospital, 222 42 Lund, Sweden; 10 Department of Endocrinology and Department of Medical and Health Sciences, Linköping University, 581 83 Linköping, Sweden; 11 Department of Internal Medicine, School of Health and Medical Sciences, Örebro University, 702 81 Örebro, SE Sweden

**Keywords:** Bilateral adrenalectomy, Cushing’s disease, Corticotroph adenoma, Nelson’s syndrome

## Abstract

**Purpose:**

Bilateral adrenalectomy (BA) still plays an important role in the management of Cushing's disease (CD). Nelson’s syndrome (NS) is a severe complication of BA, but conflicting data on its prevalence and predicting factors have been reported. The aim of this study was to determine the prevalence of NS, and identify factors associated with its development.

**Data sources:**

Systematic literature search in four databases.

**Study Selection:**

Observational studies reporting the prevalence of NS after BA in adult patients with CD.

**Data extraction:**

Data extraction and risk of bias assessment were performed by three independent investigators.

**Data synthesis:**

Thirty-six studies, with a total of 1316 CD patients treated with BA, were included for the primary outcome. Pooled prevalence of NS was 26% (95% CI 22–31%), with moderate to high heterogeneity (*I*^2^ 67%, *P* < 0.01). The time from BA to NS varied from 2 months to 39 years. The prevalence of NS in the most recently published studies, where magnet resonance imaging was used, was 38% (95% CI 27–50%). The prevalence of treatment for NS was 21% (95% CI 18–26%). Relative risk for NS was not significantly affected by prior pituitary radiotherapy [0.9 (95% CI 0.5–1.6)] or pituitary surgery [0.6 (95% CI 0.4–1.0)].

**Conclusions:**

Every fourth patient with CD treated with BA develops NS, and every fifth patient requires pituitary-specific treatment. The risk of NS may persist for up to four decades after BA. Life-long follow-up is essential for early detection and adequate treatment of NS.

**Supplementary Information:**

The online version contains supplementary material available at 10.1007/s11102-021-01158-z.

## Introduction

Cushing´s disease (CD) is a rare disorder associated with excess morbidity and increased mortality [1, 2]. Previously, bilateral adrenalectomy (BA) was the mainstay treatment for CD. During the last decades, however, other treatment modalities have emerged, including pituitary surgery, radiotherapy and medical treatments. Despite this, BA is still considered when other treatment options have failed to achieve remission, or when a rapid relief of hypercortisolism is necessary [3].

BA is considered to be a safe and effective treatment for CD [4], especially after the laparoscopic approach was introduced during the 1990s [5]. There are, however, significant drawbacks with BA, mainly the unavoidable chronic adrenal insufficiency, as well as the risk for Nelson’s syndrome (NS), i.e., growth of the remaining pituitary tumor and excessive production of ACTH, that may cause optic nerve or chiasmal compression and mucocutaneous hyperpigmentation [6].

The prevalence of NS varies between studies, mainly due to a lack of consensus on the definition and diagnostic criteria for the syndrome [7, 8]. Previously published studies are also inconsistent as to whether factors such as previous radiotherapy, age at BA, gender and duration of CD, may affect the risk of developing NS. Furthermore, high ACTH concentrations after BA have been suggested as a risk factor for developing NS [9–12].

Thus, the primary aim of this systematic review and meta-analysis was to estimate the prevalence of NS after BA for CD, both the total prevalence of NS as well the prevalence of NS requiring treatment with pituitary surgery and/or radiotherapy. The secondary aim was to investigate risk factors associated with development of NS.

## Methods

A systematic review and meta-analysis was conducted according to the Preferred Reporting Items for Systematic Reviews and Meta-Analyses (PRISMA) [13]. The PICO process was applied for the definition of the research question and eligibility criteria for the literature search. The protocol of this review was registered in the PROSPERO database (CRD42020163918).

### Search strategy

We searched PubMed, Embase, Cochrane Library and Web of Science on February 25^th^ 2020, with no start date restriction, for relevant articles by using the following terms: “Cushing´s syndrome” or “Cushing´s disease” or “Hypercortisolism” or “Pituitary ACTH hypersecretion” or “corticotroph tumor” or “corticotroph tumors” or “corticotroph adenoma” or “corticotroph adenomas” or “corticotropinoma” or “corticotropinomas” or “corticotrophinoma” or “corticotrophinomas” or “ACTH pituitary adenoma” or “ACTH pituitary adenomas” or “adrenocorticotropin pituitary adenoma” or “adrenocorticotropin pituitary adenomas” AND “bilateral adrenalectomy” or “bilateral adrenalectomies” or “total adrenalectomy” or “total adrenalectomies”. A detailed description of the search strategy is given in the Supplementary. Also, references of the included studies and relevant review articles were checked manually for additional articles. A new search was performed on January 12th 2021, prior submission, to identify any new publications.

### Study selection and eligibility criteria

Eligible studies were observational studies (cohort or cross-sectional studies) reporting the prevalence of NS in adult patients with CD treated with BA. Studies including only children (age < 18 years), review articles, letters, commentaries and meeting abstracts were excluded. Moreover, case reports, case-series and studies with a population of fewer than 10 cases were excluded. Also, studies written in languages other than English were not considered for inclusion.

### Data collection process and data extraction

Titles and abstracts from all identified articles were screened for eligibility and further full-text assessment by three independent investigators (EP, MP, OR). Discrepancies were resolved through discussion and consensus. Duplicate articles and studies with overlapping populations were excluded. In the latter case, the publication with the largest population, more comprehensive information on relevant clinical variables and/or lowest risk of bias was included.

Full-text assessment and data extraction were conducted independently by the same investigators as above. Data on the following predefined variables were extracted: first author, year of publication, region/hospital, study period, characteristics of the study population (number of patients, gender, follow-up, age at CD, age at BA, previous treatment with radiotherapy and/or pituitary surgery, ACTH concentrations at BA, MRI findings at CD and at BA), intervention (BA as primary or secondary treatment, remission status) and outcome (criteria for NS, number of patients with NS, age at NS, time from BA to NS, ACTH concentrations one year after BA, number of patients treated for NS, type of treatment; pituitary radiotherapy and/or pituitary surgery).

One of the studies included in the meta-analysis is our nationwide Swedish study on CD [2]. Additional clinical data, not provided in the original publication, was retrieved and used in the current analysis (Table 1).

**Table 1:** Characteristics of the included studies

Study	Country	Period	Study population n	Women n (%)	Patients with NS n (%)	Women with NS n (%)	Patients treated for NS n (%)	Follow-up–median/mean, [range] (yr)	Time from BA to NS –mean /median, [range] (yr)	Age at BA in patients with NS-median/Mean (yr)
Moore et al. [21]	USA	NA	120	89 (74)	9 (8)	8 (89)	8 (7)	8.0 [2.0–20.0]	8.0 [0.5–16.0]	NA
Scott et al. [23]	USA	1952–1976	26	19 (73)	1 (4)	1 (100)	1 (4)	8.0 [0.5–20.0]	2.0 [NA]	51.0
Nabarro et al. [22]	UK	1954–1976	32	NA	6 (19)	6 (100)	6 (19)	NA	NA	26.0
Cohen et al. [24]	USA	1951–1976	21	19 (90)	8 (38)	8 (100)	8 (38)	8.0 [1.0–20.0]	6.5 [1.5–12.0]	29.5
Jordan et al. [25]	USA	1952–1969	12	11 (92)	4 (33)	NA	4 (33)	NA	NA	NA
Barnett et al. [26]	New Zealand	1969–1980	15	13 (87)	3 (20)	1 (33)	NA	5.0 [2.0–11.0]	NA	NA
Kasperlik et al. [27]	Poland	1958–1982	50	45 (90)	14 (28)	12 (86)	10 (20)	12.0 [1.0–22.0]	4.8 [1.5–12.0]	23.6
Kelly et al. [28]	UK	1960–1980	38	NA	11 (29)	NA	7 (18)	10.0 [1.0–20.0]	6.0 [3.0–13.0]	45.0
Kuhn et al. [29]	France	NA	72	49 (68)	20 (28)	14 (70)	NA	NA	NA	NA
Grabner et al. [30]	Norway	1950–1987	80	NA	10 (13)	NA	10 (13)	12.5 [1.0–34.0]	9.5 [3.0–20.0]	NA
McCance et al. [31]	Northern Ireland	1972–1991	26	20 (77)	7 (27)	NA	7 (27)	5.3 [0.6–19.1]	NA	NA
Zeiger et al. [32]	USA	1983–1993	10	9 (90)	1 (10)	NA	NA	3.3 [NA]	NA	NA
Favia et al. [33]	Italy	1975–1991	41	NA	6 (15)	NA	4 (10)	NA, [2.0–16.0]	NA	NA
Kemink et al. [34]	Netherlands	1962–1991	48	44 (92)	8 (17)	8 (100)	NA	9.5 [1.0–30.0]	6.6 [1.5–13.0]	26.0
Misra et al. [35]	India	NA	16	10 (63)	2 (13)	1 (50)	2 (13)	NA	NA	NA
Jenkins et al. [36]	UK	1946–1993	38	NA	11 (29)	NA	10 (26)	NA	1.0 [0.3–9.5]	NA
Pereira et al. [37]	Brazil	NA	30	22 (73)	14 (47)	12 (86)	10 (33)	6.0 [2.0–21.0]	4.0 [0.9–10.0]	31.0
Imai et al. [38]	Japan	1957–1999	16	NA	4 (25)	NA	4 (25)	NA	NA	NA
Nagesser et al. [39]	Netherlands	1953–1989	44	33 (75)	10 (23)	9 (90)	NA	19.6 [1.0–41.6]	15.5 [7.0–24.0]	33.1
Hofmann et al. [40]	Germany	1997–2004	11	NA	1 (9)	NA	1 (9)	NA	NA	NA
Assié et al. [52]	France	1991–2002	53	45 (85)	21 (40)	NA	10 (19)	4.6 [0.5–13.5]	2 [0.5–7]	NA
Gil-Cardenas et al. [20]	Mexico	1990–2005	39	32 (82)	11 (28)	7 (64)	11 (28)	4.4 [1–15.7]	1.3 [0.2–2.8]	NA
Thompson et al. [41]	USA	1994–2004	35	NA	3 (9)	NA	3 (9)	3.6 [0.3–10]	NA	NA
Smith et al. [18]	USA	1995–2007	40	34 (85)	13 (33)	NA	7 (18)	5 [0.2–10.2]	NA	NA
Ding et al. [42]	China	2000–2008	34	29 (85)	6 (18)	6 (100)	6 (18)	4 [1.2–7.7]	NA	NA
Mehta et al. [19]	USA	NA	20	17 (85)	1 (5)	NA	1 (5)	5.4 [0.6–12]	0.8 [NA]	NA
Oßwald et al. [43]	Germany	1990–2013	29	NA	7 (24)	NA	7 (24)	11 [0.8–51]	4.3 [NA]	NA
Prajapati et al. [44]	India	1991–2013	12	NA	5 (42)	NA	5 (42)	6.7 [0.3–13]	2.7 [1.7–5]	NA
Espinosa-de-Los-Monteros et al. [45]	Mexico	1991–2014	10	NA	6 (60)	NA	4 (40)	NA	2.5 [2–8.5]	23
Graffeo et al. [46]	USA	1956–2015	88	64 (73)	47 (53)	34 (72)	14 (16)	NA [2–58]	3 [1–8]	35
Nankova et al. [47]	Bulgaria	1965–2016	36	NA	9 (25)	NA	NA	NA	NA	NA
Chiloiro et al. [48]	Italy	2003–2017	11	7 (64)	1 (9)	NA	1 (9)	6	NA	NA
Cohen et al. [49]	Argentina	1974–2011	13	9 (70)	6 (46)	4 (67)	4 (31)	14 [5–30]	2 [0.7–3.9]	31
Nagendra et al. [50]	India	2005–2018	14	NA	6 (43)	NA	4 (29)	NA	NA	NA
Ragnarsson et al. [2^a^]	Sweden	1987–2013	96	71 (74)	31 (32)	24 (77)	30 (31)	22 [0–53]	5.9 [0.5–39]	32.9
Sarkis et al. [51]	France	1990–2015	17	14 (82)	5 (29)	NA	NA	NA	6.1 [NA]	NA
Das et al. [53]	India	1984–2019	43	29 (67)	17 (40)	11 (64)	16 (37)	7 [1–12]	3 [NA]	31.1

*NS* nelson syndrome, *BA* bilateral adrenalectomy, *yr* years, *NA* not available/not applicable

^a^Additional data

Data are given as no. (%) unless otherwise indicated

### Risk of bias assessment

The Newcastle–Ottawa Scale [14], modified to suit the current study, was used for assessment of risk of bias of all included studies. Three investigators (EP, MP, OR) assessed the studies independently, and any disagreements were resolved by discussion. Selection, comparability and outcome were assessed through predefined criteria. All studies that provided information on NS as outcome, and/or corticotroph tumor progression, were included, and the definition as well as the treatment of NS were recorded (Table 1 and Table S1). A clear definition of NS and information on treatment were considered to be two of the most important components of the quality assessment. We considered the definition of NS to be clear when it included either a new visible pituitary tumor or progression of a pituitary tumor remnant following BA, alone, or in combination with high ACTH concentrations and/or hyperpigmentation. Detailed description of the criteria for the risk of bias assessment is provided in the Supplementary file. Studies with an overall score ≥ 5 (max overall grade 8) and a clear definition of NS, were considered to have a low risk of bias.

### Data synthesis and statistical analysis

Primary endpoints were the prevalence of NS, as well as the prevalence of pituitary-specific treatment for NS. Descriptive data are presented as median (range or interquartile range; IQR). Meta-analysis was performed by using the meta package in R (version 4.0.3) [15]. Statistical pooling was performed according to random-effects model due to the clinical heterogeneity among the included studies [16]. For all analyses, indices of heterogeneity, *I*^2^ statistics and Cochrane’s Q test, are reported. For the primary outcomes we estimated pooled prevalence with 95% confidence intervals (95% CI). Statistical significance was defined as *P* < 0.05. The possibility of publication bias was assessed by visual inspection of funnel plots as well as with the Egger’s test [17].

Sensitivity analyses were performed by excluding studies with an overall risk of bias < 5, and studies where information on diagnostic criteria for NS was lacking. By choosing the overall risk of bias < 5, all studies without adequate follow-up were also excluded (Table S2). Also, another sensitivity analysis was performed by including all studies reporting the number of patients with NS who received treatment for NS (Table 1).

Subgroup analyses were performed to investigate factors that may affect the prevalence of NS, namely pituitary radiotherapy prior to BA, prophylactic pituitary radiotherapy, overall radiotherapy (prior to BA or prophylactic), pituitary surgery (transcranial or transsphenoidal surgery) prior to BA, and BA as primary or secondary treatment. For these outcomes, we estimated relative risks (RRs), or pooled prevalence, with 95% CIs. Also, in a subgroup analysis, the prevalence (with 95% CI) of NS and treatment for NS were estimated in studies where MRI was used at diagnosis and during follow-up.

Uni- and bivariate meta-regression was used to investigate whether the prevalence of NS was influenced by median follow-up time or age at BA. The meta-analysis was performed by using the Metareg command in R. The estimated association is reported as β coefficient.

### Role of funding source

The funding source had no role in the design and conduction of the study; i.e., collection, management, analysis, and interpretation of the data; preparation, review, or approval of the manuscript; and decision to submit the manuscript for publication.

## Results

### Identification and description of included studies

**Fig. 1:**
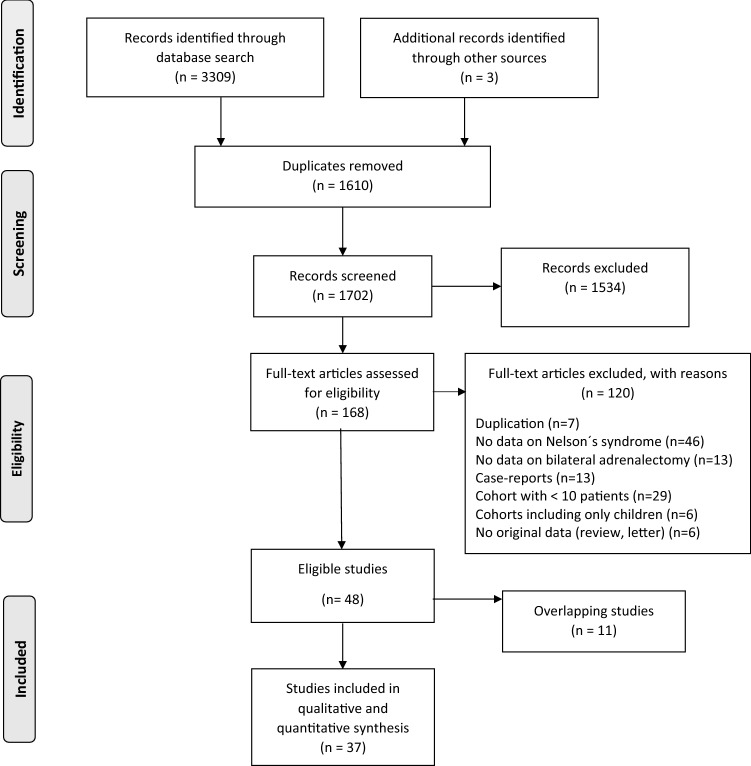
Flowchart of study selection

After removal of duplicates, 1702 articles were identified (Fig. 1). Three additional articles were found after checking the reference lists of identified articles and review papers. After reviewing titles, abstracts and full-text articles, 48 articles were considered eligible for further analysis. Of these, however, 11 articles were excluded due to overlapping or identical patient cohorts. Thus, 37 studies published between 1976 and 2020, were included in the current meta-analysis (Fig. 1). All studies had a retrospective observational design. Characteristics of the included studies are presented in Table 1. Two of the included studies had an overlapping cohort where one was used for the main outcome [18] and one [19] for the subgroup analyses on the influence of radiotherapy on the development of NS. An overview of risk of bias assessment of the eligible studies is provided in Table S2.

In total, 1316 patients with CD treated with BA were included. The median follow-up after BA was 7 years (23 studies, range 3.3–22). Median age at BA in patients with NS was 31 years (13 studies, IQR 26–34). Median time from BA to the diagnosis of NS was 4 years (19 studies) with the shortest reported time being 2 months [20] and the longest 39 years [2]. At diagnosis of NS, hyperpigmentation was reported in 155 of 188 (82%) patients (19 studies) and chiasmal compression in 24 of 129 (19%) patients [11 studies].

### Prevalence of NS

**Fig. 2:**
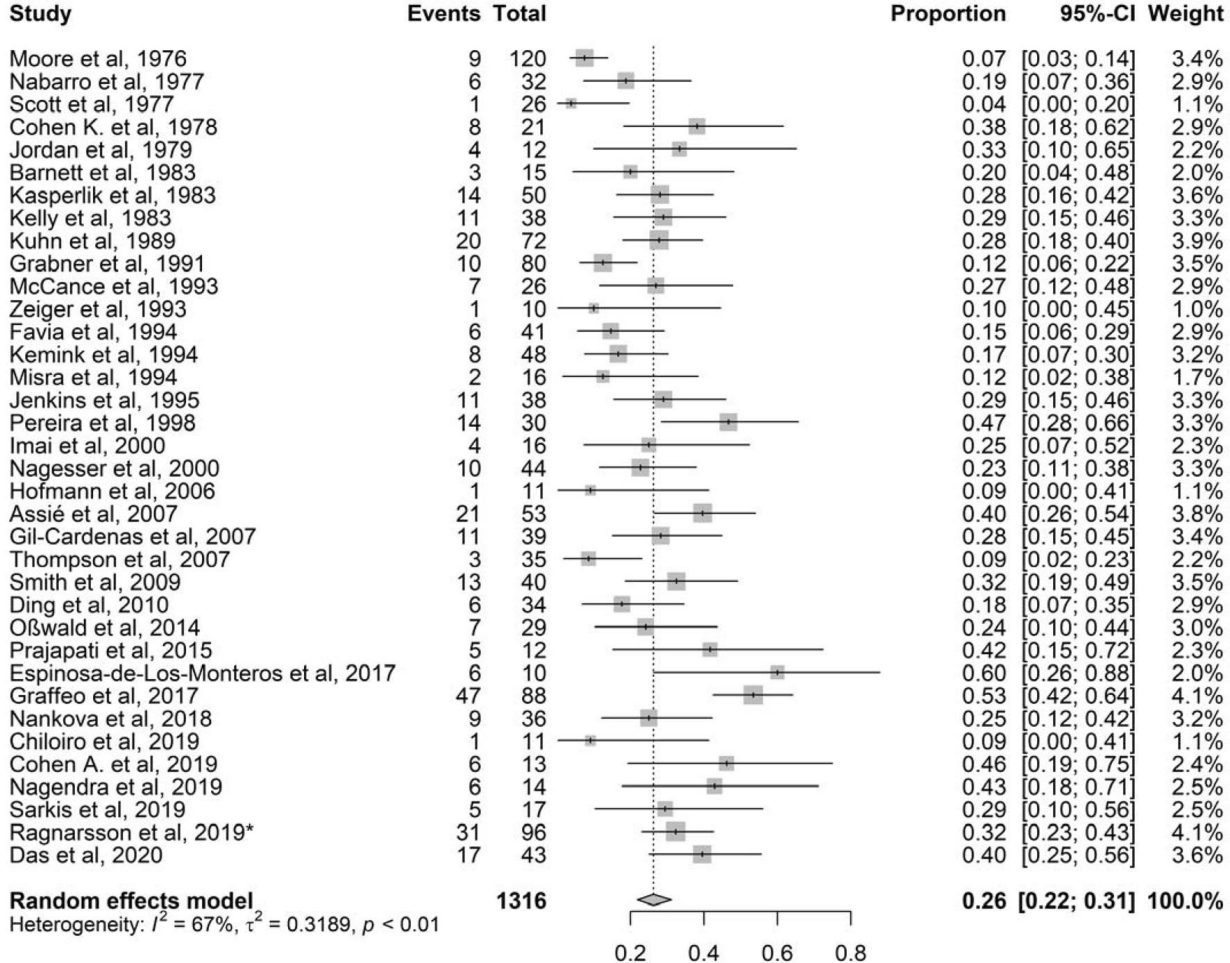
Forest plot showing individual studies and pooled prevalence of Nelson’s syndrome after bilateral adrenalectomy in patients with Cushing’s disease. *Additional data

Thirty-six of 37 studies, with total 1316 patients with CD treated with BA, were included [2, 18, 20–53]. Reported prevalence of NS ranged from 4 to 60%. The mean pooled prevalence was 26% (95% CI 22–31%) with a moderate to high heterogeneity (*I*^2^ 67%, *P* < 0.01) (Fig. 2). The Egger’s test was statistically significant (*P* = 0.01), but visual inspection showed no obvious asymmetry. The significant Egger’s test indicates publication bias, probably explained by the fact that case reports and cohorts with fewer than 10 participants were excluded (Fig. S1).

**Fig. 3:**
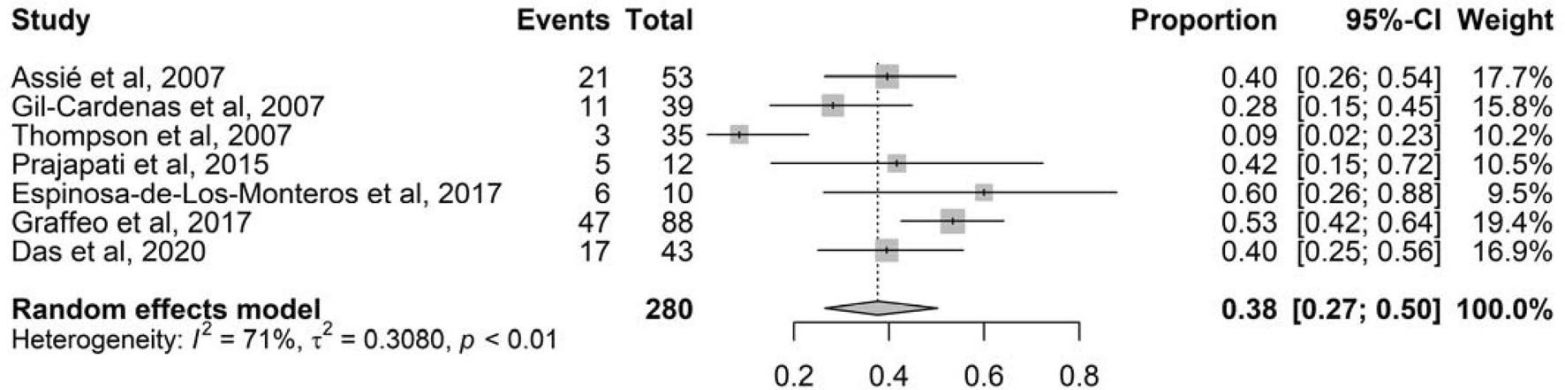
Forest plot showing individual studies using magnetic resonance imaging and pooled prevalence of Nelson’s syndrome after bilateral adrenalectomy in patients with Cushing’s disease

In a sensitivity analysis, excluding all studies with high risk of bias (overall score < 5) and no clear definition of NS, the pooled prevalence was 31% (95% CI 24–38%; *I*^2^ 76%, 17 studies, 822 patients; *P* < 0.01) (Fig. S2). In a subgroup analysis, the prevalence of NS in studies where MRI was used at diagnosis and during follow-up was 38% (Fig. 3; 95% CI 27–50%; *I*^2^ 71%, 7 studies, 280 patients; *P* < 0.01).

### Prevalence of treated NS

The pooled prevalence of treatment for NS was 21% (95% CI 18–26%; *I*^2^ 52%, *P* < 0.01) (Table 1; 29 studies with 1074 patients). Thus, the pooled prevalence was slightly lower, compared to the pooled prevalence of NS in total, as well as the heterogeneity (Fig. S3). The funnel plot showed no asymmetry and Egger’s test was not statistically significant, indicating low possibility of publication bias (Fig. S4). In a subgroup analysis, the prevalence of treated NS in studies where MRI was used at diagnosis and during follow-up was 25% (95% CI 17–35%; *I*^2^ 61%, 7 studies; *P* = 0.02).

The indication for treatment was progression of the pituitary tumor in 23 out of 28 patients (82%, five studies), optic chiasmal compression in 11 out of 91 patients (12%, 11 studies), while four patients out of 14 (one study) had both these indications for treatment. Twenty-six studies provided information on treatment modalities (pituitary surgery and/or radiotherapy). Seventy-three out of 201 patients with NS (36%) were treated with pituitary surgery, 86 (43%) with radiotherapy and 41 (20%) received both treatments.

### Radiotherapy

**Fig. 4:**
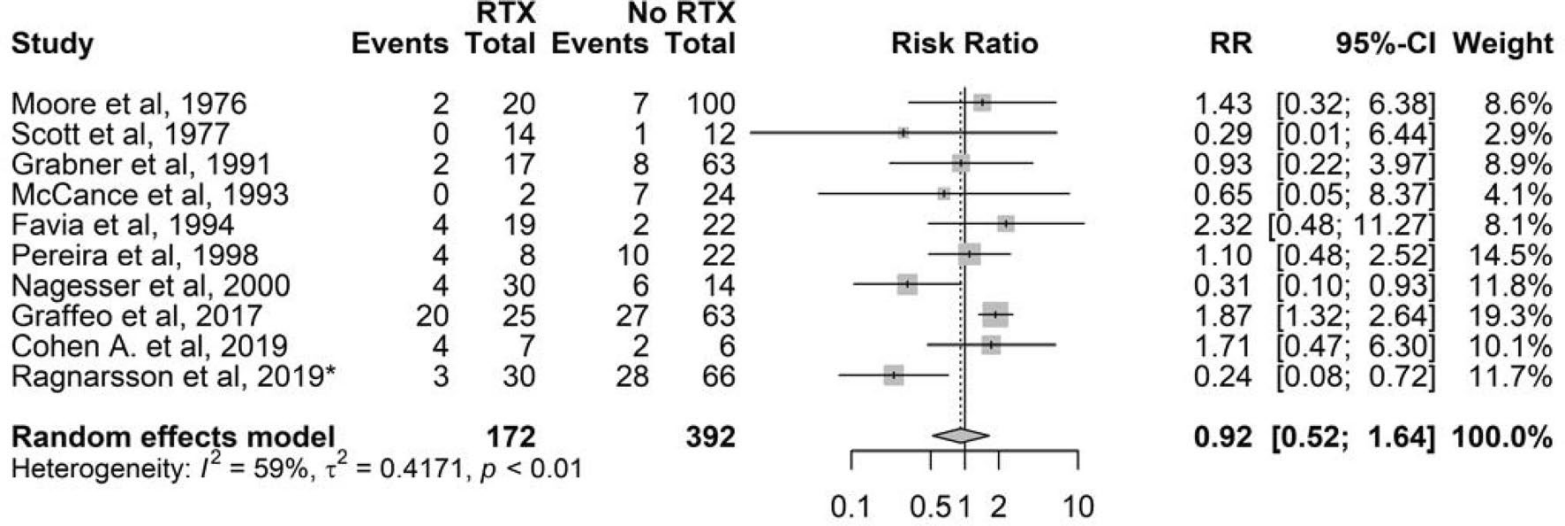
Forest plot showing the RR (relative risk) and 95% CI for Nelson’s syndrome in patients treated with radiotherapy prior to bilateral adrenalectomy versus no radiotherapy. RR could not be calculated when there were no cases in the RTX or no RTX arms, and when no events in either arm. *Additional data. RTX, radiotherapy prior to bilateral adrenalectomy or prophylactic radiotherapy

Nineteen studies provided information on radiotherapy prior to BA. However, nine studies had no events and no patients in one of the arms (radiotherapy or no radiotherapy) (Table S3). Thus, ten studies were eligible for further estimation, showing that the risk for NS in patients treated with radiotherapy prior to BA was comparable to the risk in patients not treated with radiotherapy (RR 0.9, 95% CI 0.5–1.6; 10 studies with 564 patients) (Fig. 4).

Thirteen studies provided information on prophylactic radiotherapy. However, only one study provided applicable data for calculating RR, thus subgroup analysis was not performed (Table S4). In that study [20], none of the seventeen patients who received prophylactic radiotherapy developed NS, while 11 of 22 patients without radiotherapy developed NS after a mean follow-up of 4.4 years (range 10–16 years).

By using studies with information on either previous or prophylactic radiotherapy (11 studies with 603 patients; Table S5), the pooled RR was 0.8 (95% CI 0.5–1.5).

### Pituitary surgery prior to BA

**Fig. 5:**
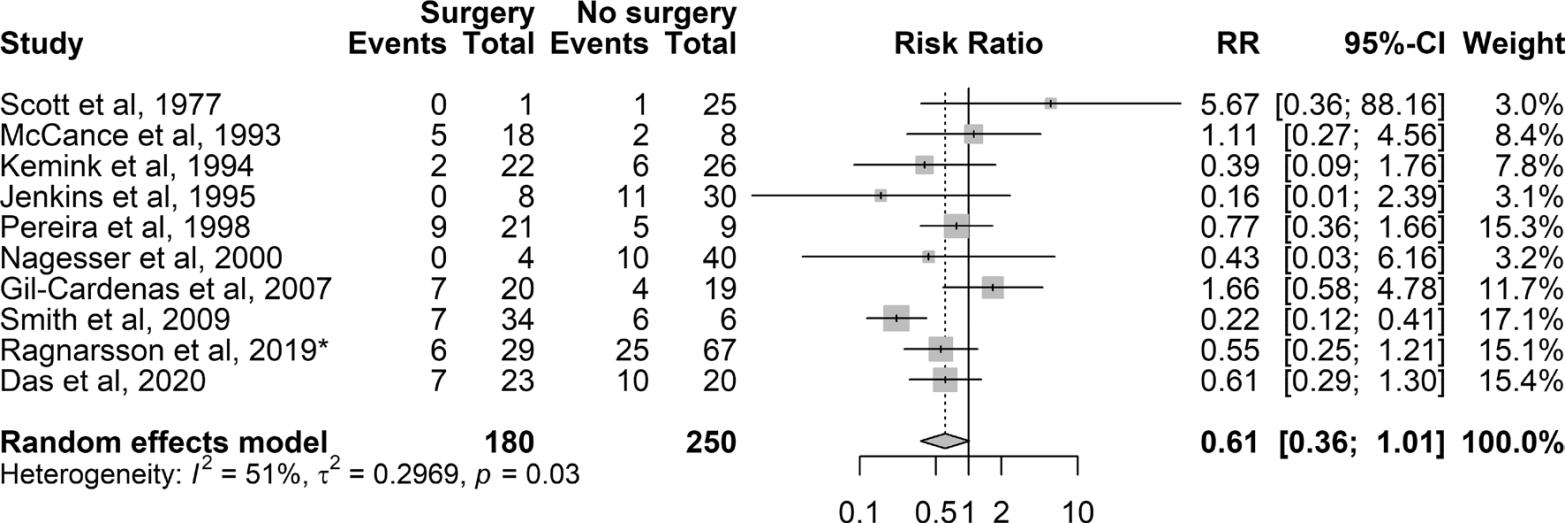
Forest plot showing the RR (relative risk) and 95% CI for Nelson’s syndrome in patients treated with pituitary surgery prior to bilateral adrenalectomy versus no pituitary surgery. RR could not be calculated when there were no cases in the surgery or no surgery arms, and when no events in either arm. *additional data. Abbreviations: Surgery, pituitary surgery prior to bilateral adrenalectomy

Of 21 studies with information on pituitary surgery prior to BA (Table S6), only ten provided information for estimation of RR. A pooled RR of 0.6 (10 studies with 430 patients; 95% CI 0.4–1.0) was found (Fig. 5), indicating that the risk for developing NS was not influenced by previous pituitary surgery.

### BA as primary or secondary treatment for CD

Information on whether patients with NS were treated primarily with BA or not, was provided in ten and nine studies, respectively (Fig. S5 and S6). The pooled prevalence of NS was 26% (95% CI 20–33%) for patients treated primarily with BA and 22% (95% CI 15–31%) for patients who had been treated with pituitary surgery and/or radiotherapy prior to BA.

### ACTH concentrations one year after BA

Four studies provided information on ACTH concentrations during the first year after BA [45, 49, 52, 53]. In a study by Assié et al. the median ACTH concentration in patients who developed NS was 301 pmol/L, compared to 79 pmol/L in patients without NS (upper range of limit; URL 13 pmol/L) [52]. The median ACTH concentration in a study by Cohen et al. was 105 pmol/L in the NS group compared to 18 pmol/L in patients without NS (*P* = 0.007) (URL 10 pmol/L) [49]. Also, in a study by Das et al., there was a statistically significant difference in ACTH concentrations one year after BA between patients with and without NS (110 vs 21 pmol/L respectively; *P* = 0.002) [53]. On the contrary, Espinosa-de-Los-Monteros et al.found no difference in ACTH concentrations between the patients with NS and those without NS [45]. Thus, three of four studies found that high ACTH concentrations one year after BA were associated with the development of NS. However, since the ACTH assays and the conditions when ACTH was collected were different in these studies (Table S7), further comparison or a meta-analysis on ACTH levels after BA was not considered feasible.

### Influence of age at BA and duration of follow-up on prevalence of NS

**Fig. 6:**
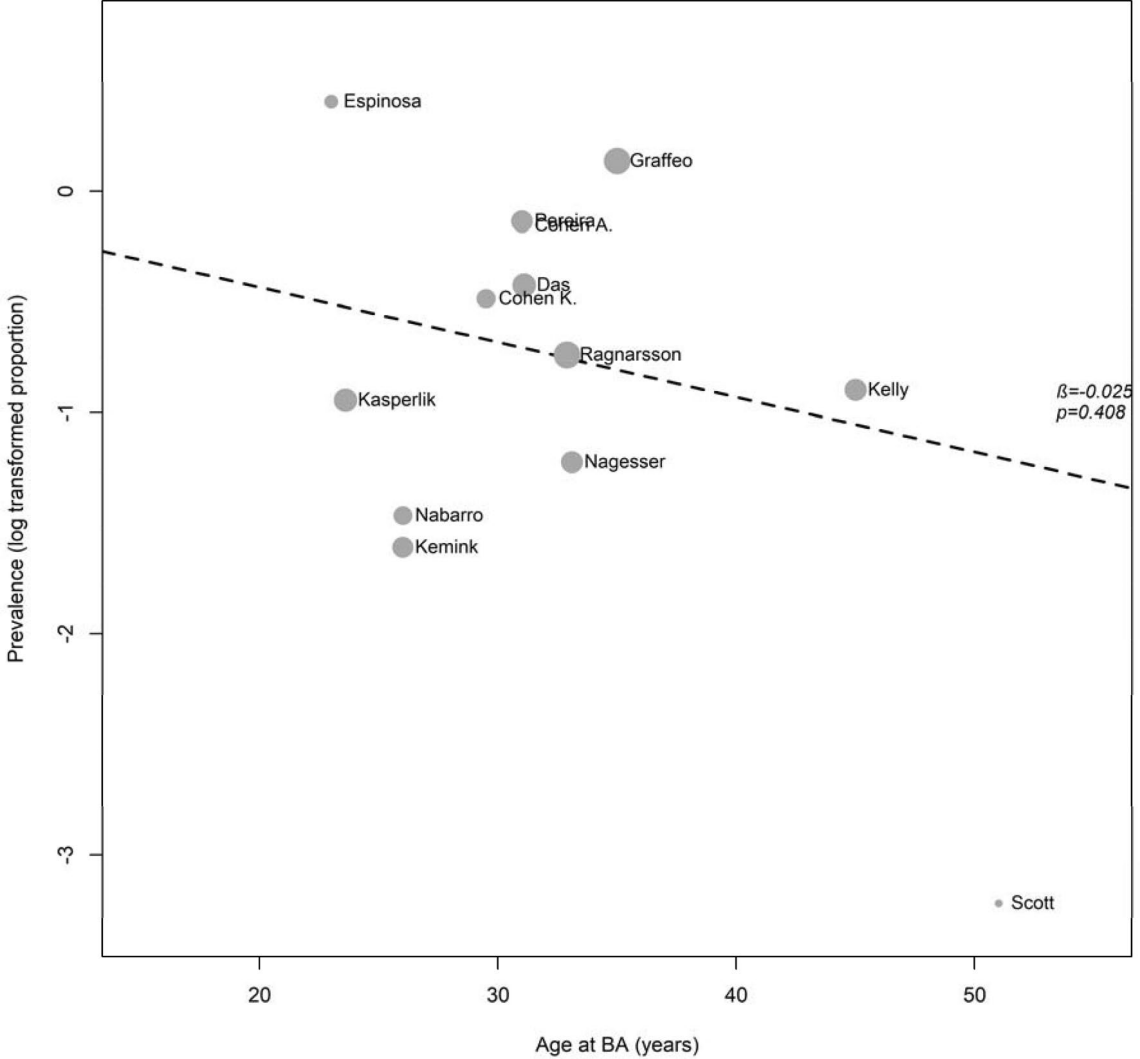
Bubble plot showing the influence of age at BA on the prevalence of Nelson’s syndrome. The bubble sizes are proportional to the weight of the studies in the meta-analysis. Coefficient estimate (β) and p value for the effect of age at BA are indicated by the regression line

In a meta-regression analysis, age at BA (β-coefficient = – 0.03, *P* = 0.4; Fig. 6) and median duration of follow-up (β-coefficient = 0.01, *P* = 0.7; Fig. S7) were not associated with prevalence of NS. After adjustment for follow-up, age at BA was still not associated with prevalence of NS (β-coefficient = -0.03, *P* = 0.4).

## Discussion

In this study we have for the first time evaluated the pooled prevalence of NS by using a meta-analysis on data from 36 studies, including more than 1300 patients with CD treated with BA. The overall prevalence of NS was 26% and the median time from BA to diagnosis of NS was 4 years, ranging from 0.2 to 39 years. The prevalence of patients requiring pituitary-specific treatment for NS was 21%. Furthermore, radiotherapy and pituitary surgery prior to BA, as well as age at BA, did not seem to affect the risk of developing NS.

Various definitions have been used for NS over the past decades [12]. Historically, the diagnosis was based on clinical findings related to mucocutaneous hyperpigmentation and chiasmal compression, together with signs of an enlarged sella turcica on skull radiography [6]. Since then, the diagnosis of NS in most studies has been based on (i) radiological evidence of a pituitary tumor that becomes visible, or a progression of a preexisting tumor, (ii) “high” ACTH concentrations, and (iii) hyperpigmentation [54]. In the studies with the highest prevalence of NS [45, 46], the diagnosis was based on rising ACTH concentrations and an expanding pituitary mass, where 2 mm increment in tumor size on MRI was considered to be a significant growth. On the contrary, the criteria for NS in studies with the lowest prevalence were based on hyperpigmentation, often but not always combined with a pituitary tumor responding to radiotherapy and/or a radiographic evidence of pituitary tumor on skull radiography [21, 23]. Thus, the great variance in the prevalence of NS between studies can, at least partly, be explained by the different definitions of NS. Consequently, in an expert opinion published in 2010, it was suggested that the diagnosis of NS should be based on an elevated level of ACTH >500 ng/L (110 pmol/L) in addition to rising levels of ACTH on at least three consecutive occasions and/or an expanding pituitary mass on MRI or CT following BA [54]. Similarly, in a recently published expert consensus recommendation, based on a systematic review, it was suggested that NS should be defined as radiological progression or new detection of a pituitary tumor on a thin-section MRI [55]. Furthermore, the authors recommend active surveillance with MRI three months after BA, and every 12 months for the first 3 years, and every 2–4 years thereafter, based on clinical findings. The meta-regression of the current analysis did not show an association between median follow-up time and prevalence of NS. Nevertheless, NS occurred as early as 2 months [20], and up to 39 years after BA [2], supporting that life-long surveillance after BA is necessary for patients with CD.

Active surveillance with MRI was more common in studies published during the last two decades. In fact, the use of MRI in recent studies resulted in earlier detection of a growing pituitary adenoma and, subsequently, contributed to a higher prevalence of NS. Namely, the seven studies including patients treated with BA after 1990 and using MRI reported higher prevalence of NS, both overall NS and treated NS.

Whether factors such as pituitary radiotherapy affects the risk for development of NS has been evaluated in several studies. Some studies have shown that radiotherapy prior to BA, or administrated prophylactically, can prevent or delay the development of NS [20, 39]. On the contrary, other studies have not demonstrated a protective effect of radiotherapy prior to BA [18, 37] and, moreover, one study found an association with tumor progression [46]. Nevertheless, the current meta-analysis indicates that radiotherapy prior to BA does not decrease the risk of developing NS. Neither did previous pituitary surgery affect the risk for NS.

Elevated ACTH concentrations during the first year after BA have been considered to be a strong predictor of NS [49, 52]. In fact, seven studies in the current analysis included cut-off levels for ACTH concentration, arbitrarily defined, for the diagnosis of NS [18, 25, 34, 36, 41, 45, 49]. Due to the different ACTH assays, and different conditions when ACTH was collected, no further analysis on ACTH levels was performed. Nevertheless, four studies [45, 49, 52, 53] reported ACTH concentrations one year after BA in both patients with and without NS. Three of these studies found that high ACTH concentrations one year after BA [49, 52, 53] were associated with pituitary tumor progression. Thus, these findings support the suggestion that ACTH should be monitored following BA in patients with CD [54, 55].

The prevalence of treatment for NS (21%), and the heterogeneity index (52%), were slightly lower than in the analysis of total prevalence of NS (26%, *I*^2^ 67%). The majority of the patients was treated with radiotherapy, followed by pituitary surgery and combination of pituitary surgery and radiotherapy. Today, surgical removal of the pituitary tumor is considered to be the first-line therapy of NS whereas radiotherapy is considered if surgery has failed or is not possible [12, 54, 56]. In a large multi-center study by Fountas et al., the 10-year progression-free survival rates after surgery alone, or with radiotherapy, for patients with NS was 80% and 81%, respectively [57]. In comparison, progression-free survival rate in patients who did not receive treatment was 51%. Reports on the efficacy of medical therapy for NS have shown inconsistent results [56].

### Strengths and limitations

This is the largest systematic review, and the first meta-analysis, on NS published to date. However, some limitations have to be acknowledged. Most important are the different diagnostic methods used to detect NS, and the different definitions of the syndrome between the studies. The majority of the studies have used the combination of hyperpigmentation, high ACTH concentrations and radiological findings for the diagnosis of NS. Notwithstanding these common criteria, there were still differences in the cut-offs of ACTH levels, the use of different radiological modalities over time as well as the radiological definition of progress of pituitary tumors. Moreover, in some studies radiological findings were used solely or in combination with either hyperpigmentation and/or bitemporal hemianopsia, ACTH concentrations or response to treatment of NS. Furthermore, in several studies a clear definition of NS was not provided. Nevertheless, we consider our attempt to address the heterogeneity of the included studies, through systematic review, quality assessment, and sensitivity and subgroup analyses to be a strength.

## Conclusions

The risk of NS after BA in patients with CD is considerable and may first become clinically evident many decades later. Thus, life-long close follow-up is necessary for an early detection of a growing pituitary tumor, and adequate treatment when needed. Although this meta-analysis did not find prior surgery or radiotherapy to be associated with risk of NS, the findings are based on a limited number of studies. Thus, in order to individualize the treatment for patients with CD, further studies are needed where these and other factors possibly associated with risk of NS are evaluated.

## Supplementary Information

Below is the link to the electronic supplementary material.

Supplementary file1 (DOCX 1208 kb)

## Data Availability

The data generated or analyzed during this study are included in this published article or in the Supplementary file.

## Abbreviations

CD: Cushing's disease
BA: Bilateral adrenalectomy
NS: Nelson’s syndrome
ACTH: Adrenocorticotropic hormone
RR: Relative risk
MRI: Magnet resonance imaging
CT: Computer tomography
