# Supplementary material for: Prevalence of Nelson’s syndrome after bilateral adrenalectomy in patients with cushing’s disease: a systematic review and meta-analysis
Source: Pituitary. 2021 May 25;24(5):797–809. doi: 10.1007/s11102-021-01158-z (PMC8416875; doi:10.1007/s11102-021-01158-z)
Supplement: Supplementary file 1 — Supplementary file1 (DOCX 1208 kb) [file 11102_2021_1158_MOESM1_ESM.docx]

**Supplemental materials**

**Table of contents**

1. Search details
2. Risk of bias assessment-Modified New Castle-Ottawa scale
3. Supplemental Tables

**Table S1.** Criteria for Nelson’s syndrome for all eligible studies

**Table S2**. Summary of risk of bias of the eligible studies based on the adjusted New Castle-Ottawa assessment

**Table S3.** Characteristics of the studies eligible for radiotherapy prior to BA

**Table S4**. Characteristics of the studies eligible for prophylactic radiotherapy

**Table S5.** Characteristics of the studies eligible for radiotherapy prior to BA or prophylactic radiotherapy

**Table S6.** Characteristics of the studies eligible for pituitary surgery prior to bilateral adrenalectomy

**Table S7.** ACTH assays and conditions for ACTH sampling for the studies reporting ACTH concentrations one year after bilateral adrenalectomy

1. Supplemental Figures

**Figure S1.** Funnel plot of prevalence of Nelson’s syndrome

**Figure S2.** Sensitivity analysis: Forest plot of prevalence of Nelson’s syndrome, excluding studies with high risk of bias (overall score <5) and with no defined criteria for Nelson’s syndrome

**Figure S3.** Forest plot of prevalence of treatment for Nelson’s syndrome

**Figure S4.** Funnel plot of prevalence of Nelson’s syndrome including only studies with information on treatment of Nelson’s syndrome

**Figure S5.** Subgroup analysis: Forest plot of prevalence of Nelson’s syndrome in patients treated with bilateral adrenalectomy as primary treatment

**Figure S6.** Subgroup analysis: Forest plot of prevalence of Nelson’s syndrome in patients treated with bilateral adrenalectomy as secondary treatment

**Figure S7.** Bubble plot showing the influence of median follow-up on the prevalence of Nelson

E. References

1. **Search details**

**Database: PubMed**

**Date:** 2019-10-04

**No of results:** 1198 ref
**Search updated 2020-02-25:** 40 new results
**2021-01-12:** 75 new results

| **Search** | **Query** | **Items found** |
| --- | --- | --- |
| [**#8**](https://www.ncbi.nlm.nih.gov/pubmed/advanced) | **Search #3 NOT #6 Filters: English** | [**1198**](https://www.ncbi.nlm.nih.gov/pubmed/?cmd=HistorySearch&querykey=8) |
| [#7](https://www.ncbi.nlm.nih.gov/pubmed/advanced) | Search #3 NOT #6 | [1498](https://www.ncbi.nlm.nih.gov/pubmed/?cmd=HistorySearch&querykey=7) |
| [#6](https://www.ncbi.nlm.nih.gov/pubmed/advanced) | Search #4 OR #5 | [4946448](https://www.ncbi.nlm.nih.gov/pubmed/?cmd=HistorySearch&querykey=6) |
| [#5](https://www.ncbi.nlm.nih.gov/pubmed/advanced) | Search animal[ti] OR animals[ti] OR rat[ti] OR rats[ti] OR mouse[ti] OR mice[ti] OR rodent[ti] OR rodents[ti] OR dog[ti] OR dogs[ti] OR cat[ti] OR cats[ti] OR hamster[ti] OR hamsters[ti] OR rabbit[ti] OR rabbits[ti] OR swine[ti] OR murine[ti] | [1836046](https://www.ncbi.nlm.nih.gov/pubmed/?cmd=HistorySearch&querykey=5) |
| [#4](https://www.ncbi.nlm.nih.gov/pubmed/advanced) | Search ((animals[mh]) NOT (animals[mh] AND humans[mh])) | [4625192](https://www.ncbi.nlm.nih.gov/pubmed/?cmd=HistorySearch&querykey=4) |
| [#3](https://www.ncbi.nlm.nih.gov/pubmed/advanced) | Search #1 AND #2 | [1530](https://www.ncbi.nlm.nih.gov/pubmed/?cmd=HistorySearch&querykey=3) |
| [#2](https://www.ncbi.nlm.nih.gov/pubmed/advanced) | Search bilateral adrenalectomy OR bilateral adrenalectomies OR total adrenalectomy OR total adrenalectomies | [4488](https://www.ncbi.nlm.nih.gov/pubmed/?cmd=HistorySearch&querykey=2) |
| [#1](https://www.ncbi.nlm.nih.gov/pubmed/advanced) | Search "cushing syndrome"[MeSH Terms] OR (Cushing*[tiab] AND (syndrome OR disease)) OR hypercortisolism OR pituitary ACTH hypersecretion OR corticotroph tumor OR corticotroph tumors OR corticotroph adenoma OR corticotroph adenomas OR corticotropinoma OR corticotropinomas OR corticotrophinoma OR corticotrophinomas OR ACTH pituitary adenoma OR ACTH pituitary adenomas OR adrenocorticotropin pituitary adenoma OR adrenocorticotropin pituitary adenomas | [29973](https://www.ncbi.nlm.nih.gov/pubmed/?cmd=HistorySearch&querykey=1) |

**Database:**  **Embase**1974 to 2019 October 03 (OvidSP)

**Date:** 2019-10-04

**No of results:** 1047 ref
**Search updated 2020-02-25:** 25 new results
**2021-01-12:** 52 new results

| **#** | **Searches** | **Results** |
| --- | --- | --- |
| 1 | exp cushing disease/ or exp cushing syndrome/ | 18927 |
| 2 | exp hypercortisolism/ | 4327 |
| 3 | exp ACTH secreting adenoma/ | 1346 |
| 4 | ((cushing$ adj3 (syndrome or disease)) or hypercortisolism or (pituitary adj3 ACTH adj3 hypersecretion) or (corticotroph adj3 tumo?r$) or (corticotroph adj3 adenoma$) or corticotropinoma$ or corticotrophinoma$ or (ACTH adj3 pituitary adj3 adenoma$) or (adrenocorticotropin adj3 pituitary adj3 adenoma$)).ab,kw,ti. | 16904 |
| 5 | 1 or 2 or 3 or 4 | 24356 |
| 6 | exp adrenalectomy/ | 20805 |
| 7 | (bilateral or total).ab,kw,ti. | 3152036 |
| 8 | 6 and 7 | 4927 |
| 9 | ((bilateral adj3 adrenalectom$) or (total adj3 adrenalectom$)).ab,kw,ti. | 2866 |
| 10 | 8 or 9 | 5437 |
| 11 | 5 and 10 | 1681 |
| 12 | (animal or animals or rat or rats or mouse or mice or rodent or rodents or dog or dogs or cat or cats or hamster or hamsters or rabbit or rabbits or swine or murine).ti. | 1954326 |
| 13 | (animal not (animal and human)).sh. | 1049897 |
| 14 | 12 or 13 | 2768560 |
| 15 | 11 not 14 | 1671 |
| **16** | **limit 15 to (english language and (embase or medline))** | **1047** |

**Database:** The Cochrane Library

**Date:** 2019-10-04

**No of results:** 11
*Cochrane trials: 11***Search updated 2020-02-25:** No new results
**2021-01-12:** 1 new result (*in Trials*)

| **ID** | **Search** | **Hits** |
| --- | --- | --- |
| #1 | MeSH descriptor: [Cushing Syndrome] explode all trees | 93 |
| #2 | MeSH descriptor: [Pituitary ACTH Hypersecretion] explode all trees | 24 |
| #3 | MeSH descriptor: [ACTH-Secreting Pituitary Adenoma] explode all trees | 17 |
| #4 | (((cushing* near/3 (syndrome or disease)) or hypercortisolism or (pituitary near/3 ACTH near/3 hypersecretion) or (corticotroph near/3 tumo*r*) or (corticotroph near/3 adenoma*) or corticotropinoma* or corticotrophinoma* or (ACTH near/3 pituitary near/3 adenoma*) or (adrenocorticotropin near/3 pituitary near/3 adenoma*))):ti,ab,kw (Word variations have been searched) | 612 |
| #5 | #1 OR #2 OR #3 OR #4 | 615 |
| #6 | (((bilateral near/3 adrenalectom*) or (total near/3 adrenalectom*))):ti,ab,kw (Word variations have been searched) | 25 |
| #7 | MeSH descriptor: [Adrenalectomy] explode all trees | 76 |
| #8 | (bilateral OR total):ti,ab,kw (Word variations have been searched) | 280785 |
| #9 | #7 AND #8 | 18 |
| #10 | #6 OR #9 | 34 |
| **#11** | **#5 AND #10** | **11** |

**Database:** Web of Science Core Collection

**Date:** 2019-10-04

**No of results:** 785
**Search updated 2020-02-25:** 21 new results
**2021-01-12:** 54 new results

| **Set** | **Results** | **Save History / Create AlertOpen Saved History** |
| --- | --- | --- |
| **# 4** | [**785**](http://apps.webofknowledge.com/summary.do?product=WOS&doc=1&qid=9&SID=F5MqKOKBTxhR9lURq6D&search_mode=CombineSearches&update_back2search_link_param=yes) | **#2 AND #1 Refined by: LANGUAGES: ( ENGLISH )** *Indexes=SCI-EXPANDED, SSCI, A&HCI, CPCI-S, CPCI-SSH, BKCI-S, BKCI-SSH, ESCI Timespan=All years* |
| # 3 | [862](http://apps.webofknowledge.com/summary.do?product=WOS&doc=1&qid=8&SID=F5MqKOKBTxhR9lURq6D&search_mode=CombineSearches&update_back2search_link_param=yes) | #2 AND #1 *Indexes=SCI-EXPANDED, SSCI, A&HCI, CPCI-S, CPCI-SSH, BKCI-S, BKCI-SSH, ESCI Timespan=All years* |
| # 2 | [2,715](http://apps.webofknowledge.com/summary.do?product=WOS&doc=1&qid=7&SID=F5MqKOKBTxhR9lURq6D&search_mode=GeneralSearch&update_back2search_link_param=yes) | TOPIC: (bilateral adrenalectomy OR bilateral adrenalectomies OR total adrenalectomy OR total adrenalectomies) *Indexes=SCI-EXPANDED, SSCI, A&HCI, CPCI-S, CPCI-SSH, BKCI-S, BKCI-SSH, ESCI Timespan=All years* |
| # 1 | [15,209](http://apps.webofknowledge.com/summary.do?product=WOS&doc=1&qid=4&SID=F5MqKOKBTxhR9lURq6D&search_mode=GeneralSearch&update_back2search_link_param=yes) | TOPIC: ((Cushing* AND (syndrome OR disease)) OR hypercortisolism OR pituitary ACTH hypersecretion OR corticotroph tumor OR corticotroph tumors OR corticotroph adenoma OR corticotroph adenomas OR corticotropinoma OR corticotropinomas OR corticotrophinoma OR corticotrophinomas OR ACTH pituitary adenoma OR ACTH pituitary adenomas OR adrenocorticotropin pituitary adenoma OR adrenocorticotropin pituitary adenomas) *Indexes=SCI-EXPANDED, SSCI, A&HCI, CPCI-S, CPCI-SSH, BKCI-S, BKCI-SSH, ESCI Timespan=All years* |

1. **Risk of bias assessment-Modified Newcastle-Ottawa scale**

| **Selection**  1. Representativeness of source population |
| --- |
| a) consecutive patients with confirmed Cushing’s disease treated with total bilateral  adrenalectomy * |
| b) no description of the derivation of the cohort |
| c) selected group with only radiotherapy as initial therapy before bilateral adrenalectomy |
|  |
| 2. Selection of the non exposed cohort |
| Non applicable |
|  |
| 3. Ascertainment of exposure |
| a) remission 100% after bilateral adrenalectomy * |
|  |
| 4. Demonstration that the outcome of interest was not present at start of the study |
| a) information that no patient with macroadenoma and chiasmal compression at bilateral |
| adrenalectomy was included *  **Comparability**   \| 1) Comparability of cohorts on the basis of the design or analysis \| \| --- \| \| a) study controls for radiotherapy * \| \| b) study controls for additional predicting factors * \| \| **Outcome** \| \| 1) Assessment of outcome \| \| a) patients with Nelson’s syndrome received treatment due to chiasmal compression * \| \| b) patients with Nelson’s syndrome received treatment * \| \|  \| \| 2) Was follow-up long enough for outcomes to occur \| \| a) yes (adequate if median/mean follow-up ≥3 years and/or minimum follow-up ≥ 1  year) * \| \|  \| \| 3) Adequacy of follow-up of cohorts \| \| a) yes * \| |

1. **Supplementary tables**

**Table S****1.** Criteria for Nelson’s syndrome for all eligible studies

| Study | Criteria for Nelson’s syndrome |
| --- | --- |
| Moore *et al,* 1976 (1) | Radiographic evidence (sella turcica, X rays) of a pituitary tumor (prior no detectable) associated with progressive cutaneous melanosis after BA |
| Scott *et al,*  1977 (2) | Pigmentation and expanding pituitary tumor thar responded to pituitary irradiation |
| Nabarro *et al,*1977 (3) | Comprises skin pigmentation, raised plasma ACTH, radiological evidence of pituitary fossa enlargement and visual field/oculomotor defect |
| Cohen K. *et al,* 1978 (4) | Hyperpigmentation, erosion or enlargement of sella turcica (X rays), visual field abnormalities, marked elevations of ACTH |
| Jordan *et al,* 1979 (5) | Hyperpigmentation, ACTH >1000 pg/ml and radiologic evidence of a pituitary tumor |
| Barnett *et al,* 1983 (6) | Hyperpigmentation, very high ACTH levels and increasing pituitary fossa size |
| Kasperlik *et al,* 1983 (7) | Hyperpigmentation and radiological evidence of pituitary tumor or defects of visual fields |
| Kelly *et al,* 1983 (8) | Increased hyperpigmentation and further pituitary expansion |
| Kuhn *et al,* 1989 (9) | Skin hyperpigmentation and increase in sella turcica volume; in three cases occurrence of optic chiasmal compression |
| Grabner *et al,* 1991 (10) | No exact definition, increased hyperpigmentation and pituitary tumors |
| McCance *et al,* 1993 (11) | Combination of hyperpigmentation, persistently elevated plasma ACTH and abnormalities in CT pituitary |
| Zeiger *et al,* 1993 (12) | No information on criteria for NS |
| Favia *et al,* 1994 (13) | Unclear definition on criteria for NS |
| Kemink *et al,* 1994 (14) | Presence of fasting ACTH >200pmol/L, hyperpigmentation of the skin and radiological evidence of a pituitary tumor >1 cm |
| Misra *et al,* 1994 (15) | Neurological or definite radiological evidence of pituitary tumor (CT) |
| Jenkins *et al,* 1995 (16) | Clinical pigmentation and a plasma ACTH value over 200ng/L 120 min after the usual hydrocortisone dose regardless of an overt pituitary tumor demonstration |
| Pereira *et al,* 1998 (17) | Presence of enlarging pituitary tumor associated with elevated fasting plasma ACTH and hyperpigmentation in CD after BA |
| Imai *et al,* 2000 (18) | No information on criteria for NS; information on treatment for NS |
| Nagesser *et al,* 2000 (19) | Full-blown NS: elevated ACTH with hyperpigmentation and expanding adenoma with visual disturbances; beginning NS: ACTH, hyperpigmentation but with microadenoma |
| Hofmann *et al,* 2006 (20) | No information on criteria for NS; information on treatment for NS |
| Assié *et al,* 2007 (21) | Corticotroph tumor progression as the occurrence of an adenoma in cases in which no adenoma was visible in previous MRI or by progression |
| Gil-Cardenas *et al,* 2007 (22) | Skin hyperpigmentation, high ACTH, presence of pituitary tumor in no preexisting or growth of a known pituitary tumor |
| Thompson *et al,* 2007 (23) | 1.Growing residual pituitary adenoma 2. ACTH >300 pg/ml and 3. Hyperpigmentation after BA |
| Smith *et al,* 2009 (24) | Elevated ACTH >200 ng/ml at any point at follow-up used as a marker for NS (although not all had clinical NS) and progressively enlarging pituitary adenomas |
| Ding *et al,* 2010 (25) | ACTH >200 ng/L indicator for NS. Clinical NS hyperpigmentation; two had bilateral hemianopsia |
| Mehta *et al,* 2013  (26) | >10% tumor growth on MRI compared to the original volume |
| Oßwald *et al,* 2014 (27) | Growth of an adenoma that had led to additional treatments (TSS, radiotherapy) |
| Prajapati *et al,* 2015 (28) | Growing residual tumor, ACTH elevated, hyperpigmentation |
| Espinosa-de-Los-Monteros *et al,* 2017 (29) | Expand­ing pituitary mass compared to pre-adrenalectomy images and plasma ACTH concentration >200 pg/mL in addi­tion to >30% increase in ACTH on at least 3 consecutive measurements |
| Graffeo *et al,* 2017 (30) | At least 2 mm of tumor growth in 1 dimension after BA in comparison with previous MRI |
| Nankova *et al,* 2018 (31) | Simultaneous presence of expanding pituitary tumor compared to pre-adrenalectomy images, persistent significantly elevated plasma ACTH, hyperpigmentation |
| Chiloiro *et al,* 2019 (32) | No information on criteria for NS; information on treatment for NS |
| Cohen A. *et al,* 2019 (33) | ACTH >500vng/L with progressive elevation (>30%) on at least 3 consecutive occasions and/or expanding lesion after BA |
| Nagendra *et al,* 2019 (34) | No information on criteria for NS |
| Ragnarsson *et al,* 2019* (35) | Patients who received treatment for NS |
| Sarkis *et al,* 2019 (36) | No information on criteria for NS |
| Das *et al,* 2020 (37) | Expanding (2 mm increase from baseline) or newly appearing pituitary adenoma ± an 08.00 h ACTH value exceeding 500 pg/ml, prior to steroid administration |

Abbreviations: NS Nelson syndrome, CD Cushing’s disease, BA bilateral adrenalectomy, ACTH Adrenocorticotropic hormone, MRI magnet resonance imaging, CT computer tomography

*additional data

**Table S2**. Summary of risk of bias of the eligible studies based on the modified Newcastle-Ottawa Scale

|  |  | **SELECTION** |  |  | **COMPARABILITY** |  | **OUTCOME** |  | **OVERALL** |
| --- | --- | --- | --- | --- | --- | --- | --- | --- | --- |
| STUDY  (Author,Year) | Representativeness of source population^1^ | Selection of the non exposed cohort^2^ | Ascertainment of exposure^3^ | Demonstration that the outcome of interest was not present at start of the study^4^ | Comparability of cohorts on the basis of the design or analysis^5^ | Assessment of outcome^6^ | Was follow-up long enough for outcomes to occur^7^ | Adequacy of follow-up of cohorts^8^ | (max score 8) |
| Assié *et al*, 2007 (21) | * | N/A | * | * | ** | * | * | * | 8 |
| Barnett *et al*, 1983 (6) | 0 | N/A | * | 0 | * | 0 | * | * | 4 |
| Chiloiro *et al*, 2019 (32) | * | N/A | * | 0 | 0 | 0 | * | * | 4 |
| Cohen A. *et al*, 2019 (33) | * | N/A | 0 | 0 | ** | * | * | * | 6 |
| Cohen K *et al*., 1978 (4) | * | N/A | 0 | 0 | * | * | * | * | 5 |
| Das *et al*, 2020 (37) | * | N/A | 0 | * | * | * | * | * | 6 |
| Ding *et al*, 2010 (25) | * | N/A | * | 0 | 0 | * | * | 0 | 4 |
| Espinosa-de-Los-Monteros *et al*, 2017 (29) | * | N/A | * | 0 | * | * | 0 | 0 | 4 |
| Favia *et al*, 1994 (13) | * | N/A | * | 0 | 0 | * | * | * | 5 |
| Gil-Gardenas *et al*, 2007 (22) | 0 | N/A | * | 0 | ** | * | * | * | 6 |
| Grabner *et al*, 1991 (10) | * | N/A | 0 | 0 | * | * | * | * | 5 |
| Graffeo *et al*, 2017 (30) | * | N/A | 0 | * | ** | * | * | * | 7 |
| Hofmann *et al*, 2006 (20) | * | N/A | 0 | * | 0 | * | 0 | * | 4 |
| Imai *et al*, 2000 (18) | * | N/A | * | 0 | 0 | * | 0 | 0 | 3 |
| Jenkins *et al*, 1995 (16) | * | N/A | 0 | 0 | * | * | 0 | * | 4 |
| Jordan *et al*, 1979 (5) | * | N/A | 0 | 0 | 0 | * | 0 | * | 3 |
| Kasperlik *et al*, 1983 (7) | * | N/A | 0 | 0 | * | * | * | * | 5 |
| Kelly *et al*, 1983 (8) | * | N/A | * | 0 | * | * | * | * | 6 |
| Kemink *et al*, 1994 (14) | * | N/A | 0 | 0 | * | 0 | * | * | 4 |
| Kuhn *et al*, 1989 (9) | 0 | N/A | 0 | 0 | * | 0 | 0 | * | 2 |
| McCance *et al*, 1994 (11) | * | N/A | * | 0 | * | * | * | * | 6 |
| Mehta *et al*, 2013 (26) | 0 | N/A | 0 | * | * | * | * | * | 5 |
| Misra *et al*, 1994 (15) | 0 | N/A | * | 0 | 0 | * | 0 | * | 3 |
| Moore *et al*, 1976 (1) | 0 | N/A | 0 | 0 | ** | * | * | * | 5 |
| Nabarro *et al*, 1977 (3) | * | N/A | 0 | 0 | * | * | 0 | * | 4 |
| Nagendra *et al*, 2019 (34) | * | N/A | 0 | 0 | 0 | * | 0 | * | 3 |
| Nagesser *et al*, 2000 (19) | * | N/A | 0 | 0 | ** | 0 | * | * | 5 |
| Nankova *et al*, 2018 (31) | * | N/A | 0 | 0 | 0 | 0 | 0 | * | 2 |
| Oßwald *et al*, 2014 (27) | * | N/A | * | 0 | 0 | * | * | * | 5 |
| Pereira *et al*, 1998 (17) | 0 | N/A | 0 | 0 | ** | * | * | * | 5 |
| Prajapati *et al*, 2015 (28) | * | N/A | 0 | 0 | * | * | * | * | 5 |
| Ragnarsson *et al*, 2019¶ (35) | * | N/A | * | 0 | 0 | * | * | * | 5 |
| Sarkis *et al*, 2019 (36) | 0 | N/A | * | 0 | 0 | 0 | * | 0 | 2 |
| Scott *et al*, 1977 (2) | * | N/A | 0 | 0 | 0 | * | * | * | 4 |
| Smith *et al*, 2009 (24) | * | N/A | * | 0 | ** | * | * | * | 7 |
| Thompson *et al*, 2007 (23) | * | N/A | 0 | 0 | 0 | * | * | 0 | 3 |
| Zeiger *et al*, 1993 (12) | * | N/A | * | 0 | 0 | 0 | 0 | 0 | 2 |

¶ additional data

| **1. Representativeness of source population (max 1 star)** |
| --- |
| a) consecutive patients with confirmed CD treated with total bilateral adrenalectomy * |
| b) no description of the derivation of the cohort |
| c) selected group with only radiotherapy as initial therapy before bilateral adrenalectomy |
| **2. Selection of the non exposed cohort (0 star)** |
| Non applicable |
| **3. Ascertainment of exposure (max 1 star)** |
| a) remission 100% after bilateral adrenalectomy * |
| **4. Demonstration that the outcome of interest was not present at start of the study (max 1 star)** |
| a) information that no patient with macroadenoma and chiasmal compression at bilateral adrenalectomy  was included * |
| **5. Comparability of cohorts on the basis of the design or analysis (max 2 stars)** |
| a) study controls for radiotherapy * |
| b) study controls for additional predicting factors * |
| **6. Assessment of outcome (max 1 star)** |
| 1. patients with NS received treatment due to chiasmal compression * 2. patients with NS received treatment * |
|  |
| **7. Was follow-up long enough for outcomes to occur (max 1 star)** |
| a) yes (adequate if median/mean follow-up ≥3 yrs and/or minimum follow-up ≥ 1 yr) * |
| **8. Adequacy of follow-up of cohorts (max 1 star)** |
| a) yes * |

**Table S3.** Characteristics of the studies eligible for radiotherapy prior to bilateral adrenalectomy

| Study | Publication  Year | Patients with NS with RTX before BA | Patients without NS with RTX before BA | Total patients with RTX (NS and no NS) | Patients with NS without RTX before BA | Patients without NS without RTX before BA | Total patients without RTX (NS and no NS) |
| --- | --- | --- | --- | --- | --- | --- | --- |
| Moore *et al*, 1976 (1) | 1976 | 2 | 18 | 20 | 7 | 93 | 100 |
| Scott *et al*, 1977 (2) | 1977 | 0 | 14 | 14 | 1 | 11 | 12 |
| Cohen K. *et al*, 1978 (4) | 1978 | 0 | 0 | 0 | 8 | 13 | 21 |
| Barnett *et al*, 1983 (6) | 1983 | 0 | 0 | 0 | 3 | 12 | 15 |
| Kelly *et al*, 1983 (8) | 1983 | 0 | 0 | 0 | 11 | 27 | 38 |
| Grabner *et al*, 1991 (10) | 1991 | 2 | 15 | 17 | 8 | 55 | 63 |
| McCance *et al*, 1993 (11) | 1993 | 0 | 2 | 2 | 7 | 17 | 24 |
| Favia *et al*, 1994 (13) | 1994 | 4 | 15 | 19 | 2 | 20 | 22 |
| Kemink *et al*, 1994 (14) | 1994 | 0 | 0 | 0 | 8 | 40 | 48 |
| Pereira *et al*, 1998 (17) | 1998 | 4 | 4 | 8 | 10 | 12 | 22 |
| Nagesser *et al*, 2000 (19) | 2000 | 4 | 26 | 30 | 6 | 8 | 14 |
| Hofmann *et al*, 2006 (20) | 2006 | 0 | 0 | 0 | 1 | 10 | 11 |
| Assié *et al*, 2007 (21) | 2007 | 0 | 0 | 0 | 21 | 32 | 53 |
| Gil-Cardenas *et al*, 2007 (22) | 2007 | 0 | 0 | 0 | 11 | 28 | 39 |
| Mehta *et al*, 2013 (26) | 2013 | 1 | 19 | 20 | 0 | 0 | 0 |
| Prajapati *et al*, 2015 (28) | 2015 | 0 | 0 | 0 | 5 | 7 | 12 |
| Graffeo *et al*, 2017 (30) | 2017 | 20 | 5 | 25 | 27 | 36 | 63 |
| Cohen A. *et al*, 2019 (33) | 2019 | 4 | 3 | 7 | 2 | 4 | 6 |
| Ragnarsson *et al*, 2019* (35) | 2019 | 3 | 27 | 30 | 28 | 38 | 66 |

Abbreviations: NS Nelson syndrome, BA bilateral adrenalectomy, RTX radiotherapy

*additional data

**Table S4**. Characteristics of the studies eligible for prophylactic radiotherapy

| Study | Publication  Year | Patients with NS with prophylactic RTX | Patients without NS with prophylactic RTX | Total patients with prophylactic RTX (NS and no NS) | Patients with NS without prophylactic RTX | Patients without NS without No prophylactic RTX | Total patients without prophylactic RTX (NS and no NS) |
| --- | --- | --- | --- | --- | --- | --- | --- |
| Moore *et al*, 1976 (1) | 1976 | 0 | 0 | 0 | 9 | 111 | 120 |
| Cohen K. *et al*, 1978 (4) | 1978 | 0 | 0 | 0 | 8 | 13 | 21 |
| Kelly *et al*, 1983 (8) | 1983 | 0 | 0 | 0 | 11 | 27 | 38 |
| Barnett *et al*, 1983 (6) | 1983 | 3 | 12 | 15 | 0 | 0 | 0 |
| Grabner *et al*, 1991 (10) | 1991 | 0 | 0 | 0 | 10 | 70 | 80 |
| McCance *et al*, 1993 (11) | 1993 | 0 | 0 | 0 | 7 | 19 | 26 |
| Zeiger *et al*, 1993 (12) | 1993 | 0 | 0 | 0 | 1 | 9 | 10 |
| Kemink *et al*, 1994 (14) | 1994 | 0 | 0 | 0 | 8 | 40 | 48 |
| Pereira *et al*, 1998 (17) | 1998 | 0 | 0 | 0 | 14 | 16 | 30 |
| Hofmann *et al*, 2006 (20) | 2006 | 0 | 0 | 0 | 1 | 10 | 11 |
| Assié *et al*, 2007 (21) | 2007 | 0 | 0 | 0 | 21 | 32 | 53 |
| Gil-Cardenas *et al*, 2007 (22) | 2007 | 0 | 17 | 17 | 11 | 11 | 22 |
| Prajapati *et al*, 2015 (28) | 2015 | 0 | 0 | 0 | 5 | 7 | 12 |

Abbreviations: NS Nelson syndrome, BA bilateral adrenalectomy, RTX radiotherapy

**Table S5.** Characteristics of the studies eligible for radiotherapy prior to bilateral adrenalectomy or prophylactic radiotherapy.

| Study | Publication  Year | Patients with NS with RTX | Patients without NS without | Total patients with RTX (NS and no NS) | Patients with NS without RTX | Patients without NS without RTX | Total patients without RTX (NS and no NS) |
| --- | --- | --- | --- | --- | --- | --- | --- |
| Moore *et al*, 1976 (1) | 1976 | 2 | 18 | 20 | 7 | 93 | 100 |
| Scott *et al*, 1977 (2) | 1977 | 0 | 14 | 14 | 1 | 11 | 12 |
| Cohen K. *et al*, 1978 (4) | 1978 | 0 | 0 | 0 | 8 | 13 | 21 |
| Barnett *et al*, 1983 (6) | 1983 | 3 | 12 | 15 | 0 | 0 | 0 |
| Kelly *et al*, 1983 (8) | 1983 | 0 | 0 | 0 | 11 | 27 | 38 |
| Grabner *et al*, 1991 (10) | 1991 | 2 | 15 | 17 | 8 | 55 | 63 |
| McCance *et al*, 1993 (11) | 1993 | 0 | 2 | 2 | 7 | 17 | 24 |
| Zeiger *et al*, 1993 (12) | 1993 | 0 | 0 | 0 | 1 | 9 | 10 |
| Favia *et al*, 1994 (13) | 1994 | 4 | 15 | 19 | 2 | 20 | 22 |
| Kemink *et al*, 1994 (14) | 1994 | 0 | 0 | 0 | 8 | 40 | 48 |
| Assié *et al*, 2007 (21) | 2007 | 0 | 0 | 0 | 21 | 32 | 53 |
| Pereira *et al,* 1998 (17) | 1998 | 4 | 4 | 8 | 10 | 12 | 22 |
| Nagesser *et al*, 2000 (19) | 2000 | 4 | 26 | 30 | 6 | 8 | 14 |
| Hofmann *et al*, 2006 (20) | 2006 | 0 | 0 | 0 | 1 | 10 | 11 |
| Gil-Cardenas *et al*, 2007 (22) | 2007 | 0 | 17 | 17 | 11 | 11 | 22 |
| Mehta *et al*, 2013 (26) | 2013 | 1 | 19 | 20 | 0 | 0 | 0 |
| Prajapati *et al*, 2015 (28) | 2015 | 0 | 0 | 0 | 5 | 7 | 12 |
| Graffeo *et al*, 2017 (30) | 2017 | 20 | 5 | 25 | 27 | 36 | 63 |
| Cohen A. *et al*, 2019 (33) | 2019 | 4 | 3 | 7 | 2 | 4 | 6 |
| Ragnarsson *et al*, 2019* (35) | 2019 | 3 | 27 | 30 | 28 | 38 | 66 |

Abbreviations: NS Nelson syndrome, BA bilateral adrenalectomy, RTX radiotherapy

*additional data

**Table S6:** Characteristics of the studies eligible for pituitary surgery prior to bilateral adrenalectomy.

| Study | Publication  Year | Patients with NS with pituitary surgery prior BA in pat with NS | Patients without NS with pituitary surgery prior BA | Total patients with pituitary surgery (NS and no NS) | Patients with NS without pituitary surgery prior BA | Patients without NS without pituitary surgery prior BA | Total patients without pituitary surgery (NS and no NS) |
| --- | --- | --- | --- | --- | --- | --- | --- |
| Moore *et al*, 1976 (1) | 1976 | 0 | 0 | 0 | 9 | 111 | 120 |
| Scott *et al*, 1977 (2) | 1977 | 0 | 1 | 1 | 1 | 24 | 25 |
| Cohen K. *et al*, 1978 (4) | 1978 | 0 | 0 | 0 | 8 | 13 | 21 |
| Barnett *et al*, 1983 (6) | 1983 | 0 | 0 | 0 | 3 | 12 | 15 |
| Kelly *et al*, 1983 (8) | 1983 | 0 | 0 | 0 | 11 | 27 | 38 |
| Grabner *et al*, 1991 (10) | 1991 | 0 | 0 | 0 | 10 | 70 | 80 |
| McCance *et al*, 1993 (11) | 1993 | 5 | 13 | 18 | 2 | 6 | 8 |
| Zeiger *et al*, 1993 (12) | 1993 | 1 | 9 | 10 | 0 | 0 | 0 |
| Kemink *et al*, 1994 (14) | 1994 | 2 | 20 | 22 | 6 | 20 | 26 |
| Jenkins *et al*, 1995 (16) | 1995 | 0 | 8 | 8 | 11 | 19 | 30 |
| Pereira *et al*, 1998 (17) | 1998 | 9 | 12 | 21 | 5 | 4 | 9 |
| Nagesser *et al*, 2000 (19) | 2000 | 0 | 4 | 4 | 10 | 30 | 40 |
| Ding *et al*, 2010 (25) | 2010 | 6 | 28 | 34 | 0 | 0 | 0 |
| Hofmann *et al*, 2006 (20) | 2006 | 1 | 10 | 11 | 0 | 0 | 0 |
| Gil-Cardenas *et al*, 2007 (22) | 2007 | 7 | 13 | 20 | 4 | 15 | 19 |
| Smith *et al*, 2009 (24) | 2009 | 7 | 27 | 34 | 6 | 0 | 6 |
| Oßwald *et al*, 2014 (27) | 2014 | 7 | 22 | 29 | 0 | 0 | 0 |
| Prajapati *et al*, 2015 (28) | 2015 | 5 | 7 | 12 | 0 | 0 | 0 |
| Chiloiro *et al*, 2019 (32) | 2019 | 1 | 10 | 11 | 0 | 0 | 0 |
| Ragnarsson *et al*, 2019* (35) | 2019 | 6 | 23 | 29 | 25 | 42 | 67 |
| Das *et al*, 2020 (37) | 2020 | 7 | 16 | 23 | 10 | 10 | 20 |

Abbreviations: NS Nelson syndrome, BA bilateral adrenalectomy

*additional data

**Table S7.** ACTH assays and conditions for ACTH sampling for the studies reporting ACTH concentrations one year after bilateral adrenalectomy

| Study | Method | Conditions |
| --- | --- | --- |
| Espinosa-de-Los-Monteros *et al,* 2017 (29) | Immunochemistry  by an automated electrochemiluminscent assay with an analytical sensibility of 1.6 pg/mL and intra- and interassay CVs of 7.4% and 7%, respectively (DiaSorin- Liaison, Saluggia, Italy) | No information provided |
| Cohen A. *et al,* 2019 (33) | Not provided | No information provided in Methods. Refers to Barber *et al* for the diagnosis of NS in Discussion. Barber et al measured ACTH at 0800h prior to glucocorticoid administration |
| Assié *et al,* 2007 (21) | Immunoradiometric assay (ELSA-ACTH, Cis  Bio International, Gif-sur-Yvette, France | Mean of several samples (median of two samples) collected on consecutive days (mean of 1.2 d) at 0800 h, 20 h after the last administration of glucocorticoid, to optimize our evaluation of baseline plasma ACTH concentration |
| Das *et al,* 2020 (37) | Measured by ECLIA (ELECSYS  Roche Diagnostics, Germany) with intra and inter-assay CV  of 1.4–2.8% and 2.3–6.4% respectively and RIA (Biosure  technologies, Nivellis, Belgium) with intra-assay and interassay  CV of 4.7 to 8% prior to that | An 08.00 h ACTH prior  to morning steroid administration |

Abbreviations: ACTH Adrenocorticotropic hormone

1. **Supplementary figures**

**Figure S1.** Funnel plot of prevalence of Nelson’s syndrome


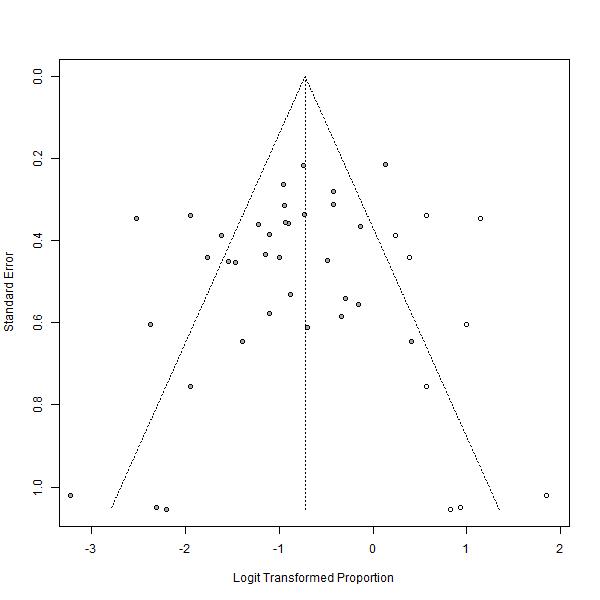


Egger’s regression test for funnel plot asymmetry: *P* = 0.01.

**Figure S2.** Sensitivity analysis: Forest plot of prevalence of Nelson’s syndrome excluding studies with high risk of bias (overall score < 5) and with no defined criteria of Nelson’s syndrome


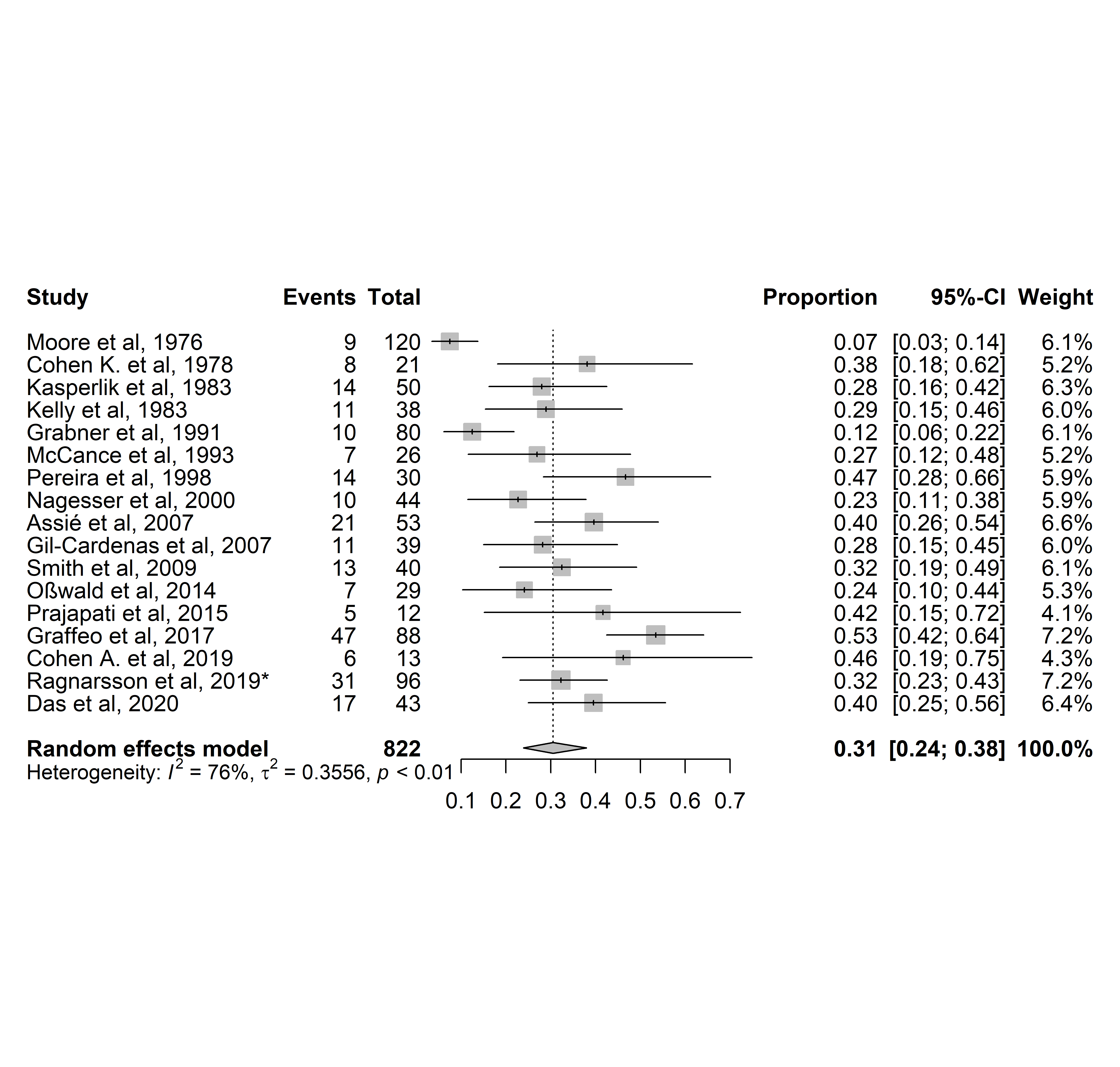


*additional data

**Figure S3.** Forest plot of prevalence of treatment for Nelson’s syndrome.


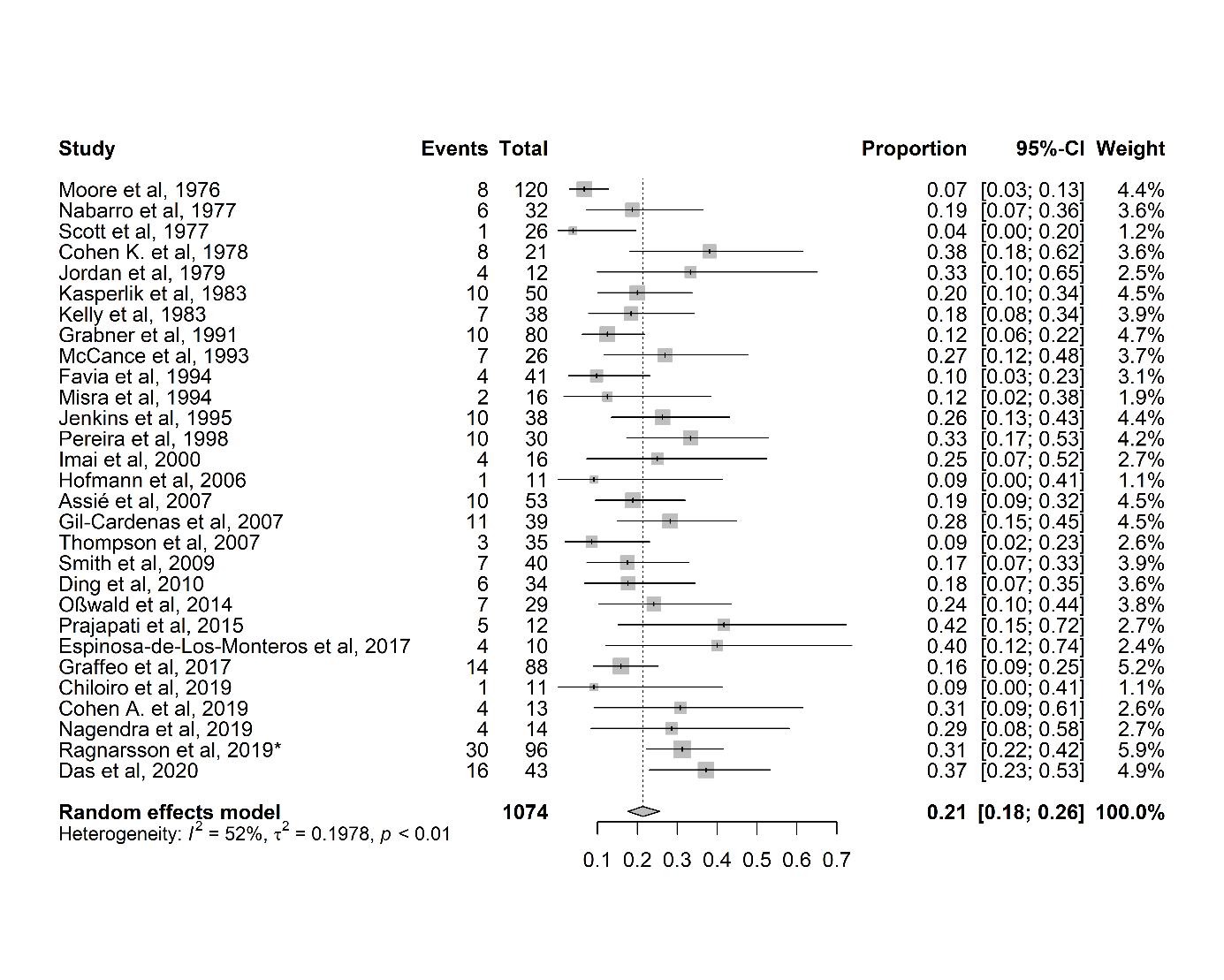


*additional data

**Figure S4.** Funnel plot of prevalence of Nelson’s syndrome including only studies with information on treatment of Nelson’s syndrome.


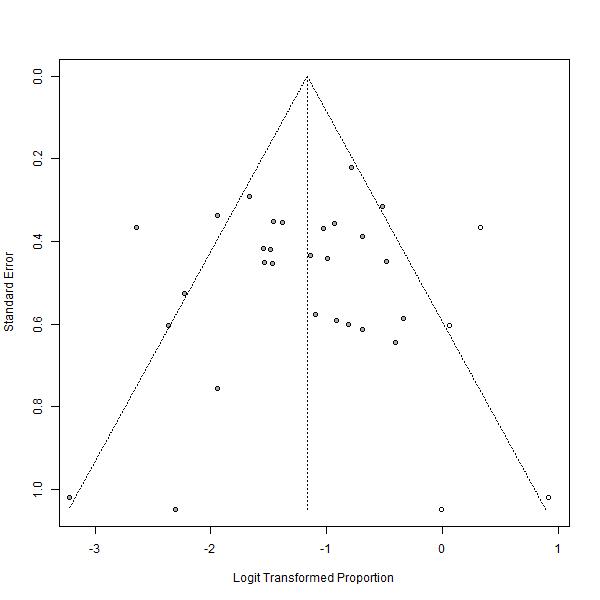
Egger’s regression test for funnel plot asymmetry: *P* = 0.2.

**Figure S5.** Subgroup analysis: Forest plot of prevalence of Nelson’s syndrome in patients treated with bilateral adrenalectomy as primary treatment.


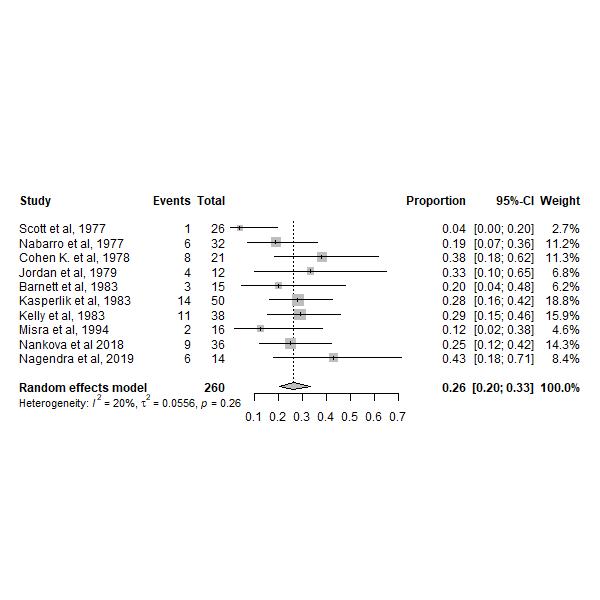


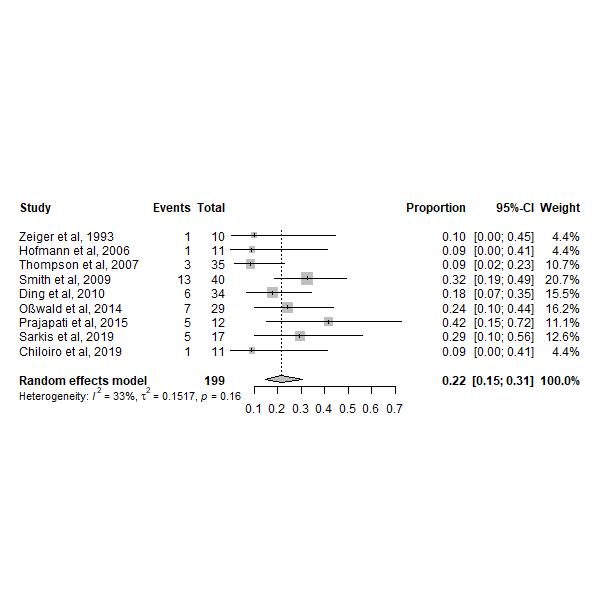
**Figure S6.** Subgroup analysis: Forest plot of prevalence of Nelson’s syndrome in patients treated with bilateral adrenalectomy as secondary treatment.

**Figure S7.** Bubble plot showing the influence of median follow-up on the prevalence of Nelson


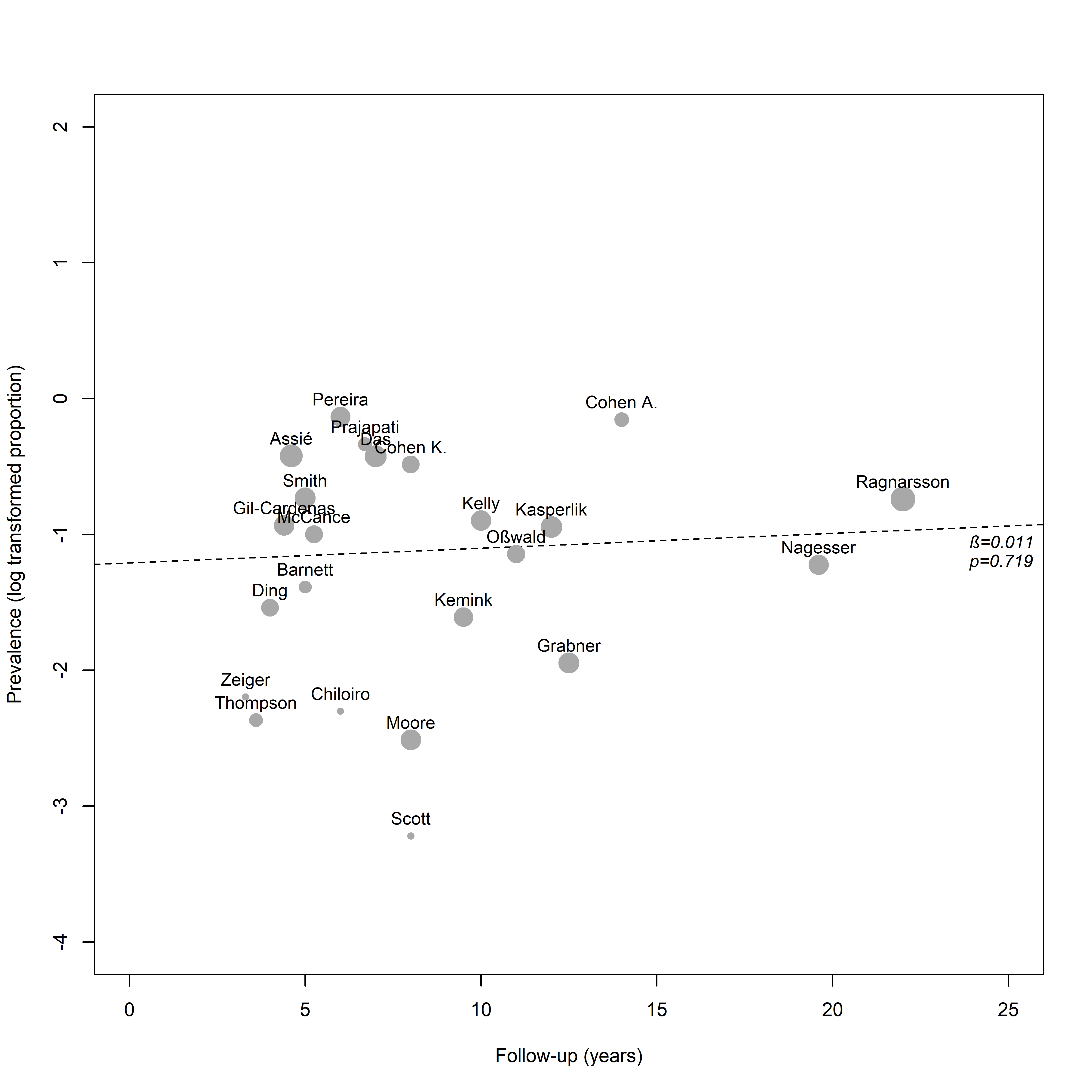


The bubble sizes are proportional to the weight of the studies in the meta-analysis.

Coefficient estimate (β) and p-value for the effect of follow-up are indicated by the regression line (italics).

**E. References**

1. Moore TJ, Dluhy RG, Williams GH, Cain JP. Nelson's syndrome: frequency, prognosis, and effect of prior pituitary irradiation. *Ann Intern Med*. 1976;85(6):731-734.

2. Scott HW, Jr., Liddle GW, Mulherin JL, Jr., McKenna TJ, Stroup SL, Rhamy RK. Surgical experience with Cushing's disease. *Annals of surgery*. 1977;185(5):524-534.

3. Nabarro JDN. ACTH secreting pituitary tumours. *Journal of the Royal College of Physicians of London*. 1977;11(4):363-375.

4. Cohen KL, Noth RH, Pechinski T. Incidence of pituitary tumors following adrenalectomy. A long-term follow-up study of patients treated for Cushing's disease. *Archives of internal medicine*. 1978;138(4):575-579.

5. Jordan RM, Cook DM, Kendall JW, Kerber CW. Nelson's syndrome and spontaneous pituitary tumor infarction. *Archives of internal medicine*. 1979;139(3):340-342.

6. Barnett AH, Livesey JH, Friday K, Donald RA, Espiner EA. Comparison of preoperative and postoperative ACTH concentrations after bilateral adrenalectomy in Cushing's disease. *Clin Endocrinol (Oxf)*. 1983;18(3):301-305.

7. Kasperlik-Zaluska AA, Nielubowicz J, Wislawski J, Hartwig W, Zaluska J, Jeske W, Migdalska B. Nelson's syndrome: incidence and prognosis. *Clin Endocrinol (Oxf)*. 1983;19(6):693-698.

8. Kelly WF, MacFarlane IA, Longson D, Davies D, Sutcliffe H. Cushing's disease treated by total adrenalectomy: long-term observations of 43 patients. *The Quarterly journal of medicine*. 1983;52(206):224-231.

9. Kuhn JM, Proeschel MF, Seurin DJ, Bertagna XY, Luton JP, Girard FL. Comparative assessment of ACTH and lipotropin plasma levels in the diagnosis and follow-up of patients with Cushing's syndrome: a study of 210 cases. *The American journal of medicine*. 1989;86(6 Pt 1):678-684.

10. Grabner P, Hauerjensen M, Jervell J, Flatmark A. Long-Term Results of Treatment of Cushings-Disease by Adrenalectomy. *Acta Chirurgica-the European Journal of Surgery*. 1991;157(8):461-464.

11. McCance DR, Russell CF, Kennedy TL, Hadden DR, Kennedy L, Atkinson AB. Bilateral adrenalectomy: low mortality and morbidity in Cushing's disease. *Clin Endocrinol (Oxf)*. 1993;39(3):315-321.

12. Zeiger MA, Fraker DL, Pass HI, Nieman LK, Cutler GB, Jr., Chrousos GP, Norton JA. Effective reversibility of the signs and symptoms of hypercortisolism by bilateral adrenalectomy. *Surgery*. 1993;114(6):1138-1143.

13. Favia G, Boscaro M, Lumachi F, D'Amico DF. Role of bilateral adrenalectomy in Cushing's disease. *World journal of surgery*. 1994;18(4):462-466.

14. Kemink L, Pieters G, Hermus A, Smals A, Kloppenborg P. Patient's age is a simple predictive factor for the development of Nelson's syndrome after total adrenalectomy for Cushing's disease. *J Clin Endocrinol Metab*. 1994;79(3):887-889.

15. Misra D, Kapur MM, Gupta DK. Incidence of Nelson's syndrome and residual adrenocortical function in patients of Cushing's disease after bilateral adrenalectomy. *The Journal of the Association of Physicians of India*. 1994;42(4):304-305.

16. Jenkins PJ, Trainer PJ, Plowman PN, Shand WS, Grossman AB, Wass JA, Besser GM. The long-term outcome after adrenalectomy and prophylactic pituitary radiotherapy in adrenocorticotropin-dependent Cushing's syndrome. *J Clin Endocrinol Metab*. 1995;80(1):165-171.

17. Pereira MA, Halpern A, Salgado LR, Mendonca BB, Nery M, Liberman B, Streeten DH, Wajchenberg BL. A study of patients with Nelson's syndrome. *Clin Endocrinol (Oxf)*. 1998;49(4):533-539.

18. Imai T, Kikumori T, Funahashi H, Nakao A. Surgical management of Cushing's syndrome. *Biomedicine & pharmacotherapy = Biomedecine & pharmacotherapie*. 2000;54 Suppl 1:140s-145s.

19. Nagesser SK, van Seters AP, Kievit J, Hermans J, Krans HM, van de Velde CJ. Long-term results of total adrenalectomy for Cushing's disease. *World journal of surgery*. 2000;24(1):108-113.

20. Hofmann BM, Fahlbusch R. Treatment of Cushing's disease: A retrospective clinical study of the latest 100 cases. *Pituitary Surgery - a Modern Approach*. 2006;34:158-184.

21. Assie G, Bahurel H, Coste J, Silvera S, Kujas M, Dugue MA, Karray F, Dousset B, Bertherat J, Legmann P, Bertagna X. Corticotroph tumor progression after adrenalectomy in Cushing's Disease: A reappraisal of Nelson's Syndrome. *J Clin Endocrinol Metab*. 2007;92(1):172-179.

22. Gil-Cardenas A, Herrera MF, Diaz-Polanco A, Rios JM, Pantoja JP. Nelson's syndrome after bilateral adrenalectomy for Cushing's disease. *Surgery*. 2007;141(2):147-151; discussion 151-142.

23. Thompson SK, Hayman AV, Ludlam WH, Deveney CW, Loriaux DL, Sheppard BC. Improved quality of life after bilateral laparoscopic adrenalectomy for Cushing's disease: a 10-year experience. *Annals of surgery*. 2007;245(5):790-794.

24. Smith PW, Turza KC, Carter CO, Vance ML, Laws ER, Hanks JB. Bilateral adrenalectomy for refractory Cushing disease: a safe and definitive therapy. *Journal of the American College of Surgeons*. 2009;208(6):1059-1064.

25. Ding XF, Li HZ, Yan WG, Gao Y, Li XQ. Role of adrenalectomy in recurrent Cushing's disease. *Chinese Medical Journal*. 2010;123(13):1658-1662.

26. Mehta GU, Sheehan JP, Vance ML. Effect of stereotactic radiosurgery before bilateral adrenalectomy for Cushing's disease on the incidence of Nelson's syndrome. *Journal of neurosurgery*. 2013;119(6):1493-1497.

27. Osswald A, Plomer E, Dimopoulou C, Milian M, Blaser R, Ritzel K, Mickisch A, Knerr F, Stanojevic M, Hallfeldt K, Schopohl J, Kuhn KA, Stalla G, Beuschlein F, Reincke M. Favorable long-term outcomes of bilateral adrenalectomy in Cushing's disease. *Eur J Endocrinol*. 2014;171(2):209-215.

28. Prajapati OP, Verma AK, Mishra A, Agarwal G, Agarwal A, Mishra SK. Bilateral adrenalectomy for Cushing's syndrome: Pros and cons. *Indian journal of endocrinology and metabolism*. 2015;19(6):834-840.

29. Espinosa-de-Los-Monteros AL, Sosa-Eroza E, Espinosa E, Mendoza V, Arreola R, Mercado M. LONG-TERM OUTCOME OF THE DIFFERENT TREATMENT ALTERNATIVES FOR RECURRENT AND PERSISTENT CUSHING DISEASE. *Endocrine practice : official journal of the American College of Endocrinology and the American Association of Clinical Endocrinologists*. 2017;23(7):759-767.

30. Graffeo CS, Perry A, Carlstrom LP, Meyer FB, Atkinson JLD, Erickson D, Nippoldt TB, Young WF, Pollock BE, Van Gompel JJ. Characterizing and predicting the Nelson-Salassa syndrome. *Journal of neurosurgery*. 2017;127(6):1277-1287.

31. Nankova A, Yaneva M, Elenkova A, Tcharaktchiev D, Marinov M, Hadzhiyanev A, Sechanov T, Gantchev G, Todorov G, Kirilov G, Kalinov K, Andreeva M, Zacharieva S. Cushing's Syndrome: A Historic Review of the Treatment Strategies and Corresponding Outcomes in a Single Tertiary Center over the Past Half-Century. *Hormone and metabolic research = Hormon- und Stoffwechselforschung = Hormones et metabolisme*. 2018;50(4):280-289.

32. Chiloiro S, Giampietro A, Raffaelli M, D'Amato G, Bima C, Lauretti L, Anile C, Lombardi CP, Rindi G, Bellantone R, De Marinis L, Pontecorvi A, Bianchi A. Synchronous bilateral adrenalectomy in ACTH-dependent hypercortisolism: predictors, biomarkers and outcomes. *Endocrine*. 2019;66(3):642-649.

33. Cohen AC, Goldney DC, Danilowicz K, Manavela M, Rossi MA, Gomez RM, Cross GE, Bruno OD. Long-term outcome after bilateral adrenalectomy in Cushing's disease with focus on Nelson's syndrome. *Archives of endocrinology and metabolism*. 2019.

34. Nagendra L, Bhavani N, Pavithran PV, Kumar GP, Menon UV, Menon AS, Kumar L, Kumar H, Nair V, Abraham N, Narayanan P. Outcomes of Bilateral Adrenalectomy in Cushing's Syndrome. *Indian journal of endocrinology and metabolism*. 2019;23(2):193-197.

35. Ragnarsson O, Olsson DS, Papakokkinou E, Chantzichristos D, Dahlqvist P, Segerstedt E, Olsson T, Petersson M, Berinder K, Bensing S, Hoybye C, Eden-Engstrom B, Burman P, Bonelli L, Follin C, Petranek D, Erfurth EM, Wahlberg J, Ekman B, Akerman AK, Schwarcz E, Bryngelsson IL, Johannsson G. Overall and Disease-Specific Mortality in Patients With Cushing Disease: A Swedish Nationwide Study. *J Clin Endocrinol Metab*. 2019;104(6):2375-2384.

36. Sarkis P, Rabilloud M, Lifante JC, Siamand A, Jouanneau E, Gay E, Chaffanjon P, Chabre O, Raverot G. Bilateral adrenalectomy in Cushing's disease: Altered long-term quality of life compared to other treatment options. *Annales d'endocrinologie*. 2019;80(1):32-37.

37. Das L, Bhansali A, Pivonello R, Dutta P, Bhadada SK, Ahuja CK, Mavuduru R, Kumar S, Behera A, Saikia UN, Dhandapani S, Walia R. ACTH increment post total bilateral adrenalectomy for Cushing's disease: a consistent biosignature for predicting Nelson's syndrome. *Pituitary*. 2020;23(5):488-497.
